# Mitochondrial dysfunction, neuroinflammation, and associated mechanisms in sepsis-associated encephalopathy: from pathogenesis to emerging therapeutics

**DOI:** 10.3389/fnins.2026.1824178

**Published:** 2026-04-14

**Authors:** Yong Shen, Xue-Mei Ye, Ping-Yang Li, Si-Lei Chen

**Affiliations:** 1 West China Hospital Sichuan University, Meishan Hospital, Meishan, China; 2 Department of the Intensive Care Unit, Meishan People’s Hospital, Meishan, China

**Keywords:** ferroptosis, microglia, mitochondrial dysfunction, neuroinflammation, NLRP3 inflammasome, sepsis-associated encephalopathy, therapeutics

## Abstract

Sepsis-associated encephalopathy (SAE) is a devastating neurological complication of sepsis, leading to diffuse brain dysfunction, long-term cognitive deficits, and increased mortality. Its pathogenesis is complex, with mitochondrial dysfunction and neuroinflammation emerging as central, interconnected drivers. This review systematically elucidates the pathogenic crosstalk between these two processes. We detail how dysregulated mitochondrial dynamics (e.g., Drp1-mediated fission), impaired biogenesis (via the proliferator-activated receptor-gamma coactivator-1α axis), oxidative stress, and the activation of mitochondria-dependent cell death pathways (ferroptosis, pyroptosis) contribute to neuronal injury. Concurrently, microglial activation, particularly through the NOD-, LRR- and pyrin domain-containing protein 3 (NLRP3) inflammasome, creates a vicious cycle that exacerbates mitochondrial damage and synaptic loss. Furthermore, we summarize emerging therapeutic strategies that target this mitochondrial-neuroinflammatory axis, including molecular hydrogen, mitochondria-targeted peptides (*SS-31*), natural compounds, and specific inhibitors (e.g., *Mdivi-1*, *MCC950*). The integration of recent insights on the gut-brain axis and cerebral metabolomics further expands the therapeutic landscape. Ultimately, targeting this core axis offers a promising paradigm for developing effective interventions to improve neurological outcomes in septic patients.

## 1 Introduction

Sepsis-associated encephalopathy (SAE) is a prevalent and devastating neurological complication of sepsis, affecting approximately 50–70% of patients in intensive care units (Li et al., 2025; Hong et al., 2023). It is associated with significantly increased in-hospital mortality, with rates ranging from 30 to 60% depending on disease severity and comorbid conditions (Li et al., 2025; Han et al., 2025). Survivors often experience long-term cognitive impairment, including deficits in memory, attention, and executive function, which profoundly diminish their quality of life and impose substantial burdens on caregivers and healthcare systems (Pan et al., 2022; Andonegui et al., 2018). Despite its clinical significance, the underlying pathophysiology of SAE remains incompletely understood, and no specific pharmacological treatment is currently available (Hong et al., 2023; Catarina et al., 2021). The diffuse cerebral dysfunction in SAE is not caused by direct central nervous system (CNS) infection but rather by a systemic inflammatory response that triggers a cascade of events within the brain.

Historically, research has focused on neuroinflammation, blood–brain barrier (BBB) disruption, and neurotransmitter imbalances (Hong et al., 2023). However, in recent years, mitochondrial dysfunction has emerged as a cornerstone of SAE pathogenesis. Mitochondria, essential for cellular energy production, calcium homeostasis, and regulation of apoptosis, are highly vulnerable to inflammatory insults (Liu et al., 2025). In SAE, mitochondrial impairment encompasses defective oxidative phosphorylation, Adenosine Triphosphate (ATP) depletion, excessive reactive oxygen species (ROS) production, altered dynamics (fission/fusion imbalance), and compromised quality control mechanisms like mitophagy (Liu et al., 2025). These defects directly contribute to bioenergetic failure, oxidative stress, and initiation of cell death pathways in neurons and glial cells.

Concurrently, neuroinflammation, predominantly mediated by activated microglia and astrocytes, amplifies brain injury (Modafferi et al., 2024). The release of pro-inflammatory cytokines (e.g., tumor necrosis factor-alpha [TNF-*α*], interleukin-1β [IL-1β], interleukin-6[IL-6]) and the activation of multiprotein complexes like the NOD-, LRR- and pyrin domain-containing protein 3 (NLRP3) inflammasome lead to synaptic damage, neuronal apoptosis, and exacerbated mitochondrial dysfunction (Ko et al., 2021). A vicious cycle ensues, where mitochondrial damage releases damage-associated molecular patterns (DAMPs) that further fuel neuroinflammation (Dela Cruz and Kang, 2018).

This review synthesizes current knowledge on the interplay between mitochondrial dysfunction and neuroinflammation in SAE. It delves into specific mechanisms—including mitochondrial dynamics, biogenesis, ferroptosis, pyroptosis, and BBB breakdown—and evaluates promising therapeutic strategies targeting these interconnected pathways, drawing from extensive *in vivo* and *in vitro* evidence.

## 2 Mitochondrial dysfunction: a central player in SAE

### 2.1 Disruption of mitochondrial dynamics

Mitochondria are highly dynamic organelles that undergo continuous cycles of fission and fusion, a process critical for maintaining their structural integrity, functional efficiency, and quality control (Nunnari and Suomalainen, 2012). In SAE, this delicate balance is profoundly disrupted, leading to a pronounced shift toward excessive fission, which is now recognized as a seminal event in the progression of neuronal injury and cognitive deficits (Nunnari and Suomalainen, 2012).

#### 2.1.1 Excessive fission mediated by dynamin-related protein 1 (Drp1)

Excessive Mitochondrial Fission is predominantly driven by the upregulation and activation of Drp1, a cytosolic GTPase that translocates to the mitochondrial outer membrane to execute fission (Kleele et al., 2021). Preclinical models of SAE, whether induced by lipopolysaccharide (LPS) or cecal ligation and puncture (CLP), consistently demonstrate increased Drp1 expression and enhanced recruitment to mitochondria in hippocampal neurons and microglia (Haileselassie et al., 2020; Zhong et al., 2023). This activation is often facilitated by post-translational modifications or interactions with adaptor proteins such as Fis1 (Mitochondrial fission 1 protein) (Wang et al., 2025). The consequence is widespread mitochondrial fragmentation, characterized by smaller, punctate mitochondria with disrupted cristae. This fragmented state is inherently dysfunctional, leading to impaired oxidative phosphorylation, collapse of the mitochondrial membrane potential (MMP), and exacerbated production of ROS. The critical role of Drp1 is further corroborated by interventional studies. Pharmacological inhibition of Drp1 with Mdivi-1 effectively attenuates mitochondrial fragmentation, restores ATP production, reduces oxidative stress, and ultimately rescues cognitive and synaptic function in murine models of SAE (Dai et al., 2025; Hong et al., 2025). Beyond neuronal damage, Drp1-mediated fission in cerebral endothelial cells compromises BBB integrity by disrupting tight junction (TJ) proteins (Hao et al., 2025). The inhibitor *P110*, which specifically blocks the Drp1-Fis1 interaction, was shown to mitigate this BBB dysfunction, highlighting the central role of this specific interaction in sepsis-induced brain injury (Haileselassie et al., 2020) (Table 1).

**Table 1:** Mechanisms of mitochondrial dysfunction in sepsis-associated encephalopathy.

Category	Key alterations	Key regulators	Functional consequences
Dynamics imbalance	Excessive fission, Impaired fusion	↑Drp1, Fis1; ↓MFN1/2, OPA1	Fragmentation, bioenergetic failure, ROS ↑
Impaired biogenesis	Suppressed PGC-1α/NRF1/2/TFAM axis	↓PGC-1α, NRF1, NRF2, TFAM	Failed mitochondrial renewal, energy crisis
Oxidative stress	ROS overload, antioxidant defense failure	mtROS, NOX; ↓Nrf2, GPX4, GSH, UCP2	Macromolecular damage, NLRP3 activation
Mitophagy dysfunction	Impaired clearance of damaged mitochondria	↓PINK1, PARKIN, BNIP3/NIX	Accumulation of dysfunctional mitochondria
Cell death pathways	Apoptosis, Pyroptosis, Ferroptosis	Bax/Bak, Omi/HtrA2, Caspase-3; NLRP3, GSDMD; GPX4↓	Neuronal and glial loss

This table summarizes the key mechanisms and consequences of mitochondrial dysfunction in SAE, including alterations in dynamics, biogenesis, oxidative stress, quality control, and cell death pathways. SAE, Sepsis-associated encephalopathy; Drp1, Dynamin-related protein 1; Fis1, Mitochondrial fission 1 protein; MFN1/2, Mitofusin 1/2; OPA1, Optic atrophy 1; PGC-1α, Peroxisome proliferator-activated receptor-gamma coactivator-1α; NRF1/2, Nuclear respiratory factor 1/2; TFAM, Mitochondrial transcription factor A; ROS, Reactive oxygen species; ETC, Electron transport chain; NOX, NADPH oxidase; Nrf2, Nuclear factor erythroid 2–related factor 2; GPX4, Glutathione peroxidase 4; GSH, Glutathione; UCP2, Uncoupling protein 2; PINK1, PTEN-induced kinase 1; BNIP3, BCL2/adenovirus E1B 19 kDa protein-interacting protein 3; NIX, Nip3-like protein X; Bax, BCL2-associated X protein; Bak, BCL2 antagonist/killer; Omi/HtrA2, Serine protease Omi/mitochondrial; NLRP3, NOD-, LRR- and pyrin domain-containing protein 3; GSDMD, Gasdermin D.

#### 2.1.2 Impaired fusion and network integrity

Impaired Mitochondrial Fusion constitutes the other facet of the imbalance. Fusion, which allows for the complementation of damaged mitochondrial components, is mediated by mitofusins (MFN1, MFN2) on the outer membrane and optic atrophy 1 on the inner membrane (von der Malsburg et al., 2023). In SAE, the expression of these fusion proteins is often downregulated. For instance, the protective effects of hydrogen (H_2_) gas have been linked to its ability to upregulate MFN2 expression, thereby promoting fusion and contributing to improved mitochondrial network integrity and function (Cui et al., 2024). The loss of fusion activity prevents the dilution of damaged components and the optimization of energy distribution across the mitochondrial network, thereby compounding the deleterious effects of excessive fission (Cui et al., 2024).

The pathological significance of disrupted mitochondrial dynamics extends beyond bioenergetic failure. A fragmented mitochondrial network is more prone to being targeted for autophagic clearance; however, when excessive, it can overwhelm the mitophagy system, leading to the accumulation of damaged organelles. Furthermore, mitochondrial fission is intimately linked to the activation of inflammatory and cell death pathways. For example, the gasdermin D (GSDMD)/Drp1 signaling pathway has been identified as a key mechanism mediating hippocampal synaptic damage and abnormalities in neural oscillations, bridging mitochondrial dysfunction to pyroptotic cell death (Fu et al., 2024). Similarly, nuclear respiratory factor 2(Nrf2) and Yes-associated protein 1(Yap1) have been shown to mitigate ferroptosis partly by maintaining mitochondrial dynamic homeostasis, suppressing the expression of fission proteins like Drp1 and Fis1 (Duan et al., 2024; Yang et al., 2024) (Table 1).

In conclusion, the disruption of mitochondrial dynamics, marked by Drp1-dominated excessive fission and compromised fusion, is a pivotal mechanism in SAE pathogenesis. It serves as a convergence point for multiple injurious processes, including bioenergetic crisis, oxidative stress, neuroinflammation, and programmed cell death, making it a promising therapeutic target for intervention.

### 2.2 Impairment of mitochondrial biogenesis

Mitochondrial biogenesis is the essential process through which cells generate new mitochondria to maintain energy homeostasis, replace damaged organelles, and adapt to increased metabolic demands. This complex process is predominantly regulated by a central signaling axis, and its impairment in SAE represents a fundamental failure of the brain’s adaptive and reparative capacity, directly contributing to bioenergetic crisis and neuronal vulnerability.

#### 2.2.1 The core regulatory axis: proliferator-activated receptor-gamma coactivator-1α (PGC-1α), nuclear respiratory factor 1/2 (NRF1/2), and mitochondrial transcription factor a (TFAM)

The master regulator of mitochondrial biogenesis is the peroxisome PGC-1α. Under physiological conditions, PGC-1α serves as a transcriptional coactivator that integrates signals from various pathways, including those involved in energy sensing and stress response. Once activated, PGC-1α coordinately upregulates the expression of several nuclear transcription factors, principally NRF1 and NRF2 (not to be confused with NF-E2-related factor 2, also abbreviated Nrf2, which is primarily involved in antioxidant response) (Meng et al., 2024). NRF1 and NRF2 then transactivate a suite of nuclear genes encoding mitochondrial proteins, including those essential for the electron transport chain (ETC) (Meng et al., 2024). Crucially, NRF1 also induces the expression of TFAM (U-Pathi et al., 2023). TFAM is imported into the mitochondrial matrix, where it plays an indispensable role in mitochondrial DNA (mtDNA) replication, transcription, and maintenance (U-Pathi et al., 2023). Therefore, the PGC-1α/NRF1/NRF2/TFAM pathway acts as the primary conduit for signaling from the nucleus to the mitochondrion, driving the synthesis of both nuclear- and mitochondrial-encoded components required for new mitochondrion formation (Table 1).

#### 2.2.2 Dysregulation and energetic failure in SAE

In the context of SAE, this finely tuned biogenetic program is significantly disrupted. The septic insult, characterized by an overwhelming inflammatory and oxidative milieu, can suppress the expression and/or activity of PGC-1α (Li et al., 2021). This suppression leads to a downstream reduction in the expression of NRF1, NRF2, and TFAM. Consequently, the synthesis of critical ETC subunits is compromised, mtDNA copy number may decline, and the overall capacity for oxidative phosphorylation is diminished (Li et al., 2021). This failure to generate new, functional mitochondria occurs precisely when energy demand is heightened due to inflammatory processes and cellular stress, creating a critical bioenergetic deficit. The resulting ATP depletion impairs essential neuronal functions, including the maintenance of synaptic plasticity and ion gradients, ultimately leading to synaptic dysfunction and cognitive decline. It is noteworthy that in certain cell types, such as astrocytes, an initial increase in mitochondrial biogenesis has been observed as a compensatory response to septic injury (Wang et al., 2014); however, this compensatory mechanism appears insufficient or becomes maladaptive over time, failing to overcome the global bioenergetic failure in the brain (Table 1).

#### 2.2.3 Therapeutic activation of biogenesis

Therapeutic activation of impaired mitochondrial biogenesis has emerged as a highly promising strategy for SAE treatment, with multiple interventions demonstrating efficacy through direct or indirect activation of this core regulatory axis. Molecular H_2_ has been shown to robustly activate the PGC-1α pathway in the brains of septic mice following inhalation (e.g., 2% concentration). This activation results in increased protein levels of NRF2 and TFAM, enhanced mitochondrial membrane potential, elevated ATP content, and ultimately improved cognitive function (Cui et al., 2024; Xie et al., 2021). Notably, H_2_’s simultaneous mitigation of oxidative stress likely creates a more favorable cellular environment for PGC-1α activation. In the realm of mitochondrial-targeted peptides, the tetrapeptide SS-31 (Elamipretide) selectively accumulates in the inner mitochondrial membrane. In SAE models, SS-31 not only indirectly facilitates biogenesis by preserving mitochondrial cristae structure and reducing ROS, but has also been demonstrated to directly support the PGC-1α pathway, promoting the synthesis of functional mitochondria and subsequently inhibiting hippocampal apoptosis and neuroinflammation (Wu et al., 2015). Furthermore, natural compounds and other agents show significant regulatory potential: *Sodium Tanshinone IIA Sulfonate* (STS), an active component of *Danshen*, ameliorates mitochondrial dysfunction and enhances synaptic plasticity by activating the SIRT1/PGC-1α/NRF1/TFAM pathway (Song et al., 2026), while inhibition of Fgr kinase attenuates SAE through the SIRT1/PGC-1α signaling pathway (Liu et al., 2023). These findings collectively underscore the pivotal role of upstream regulators like SIRT1 in activating mitochondrial biogenesis, highlighting multiple therapeutic avenues for addressing mitochondrial impairment in SAE (Table 1).

Beyond pharmacological activation of endogenous biogenesis, intercellular mitochondrial transfer has recently emerged as a revolutionary therapeutic strategy for restoring mitochondrial function in damaged cells (Kan et al., 2025; Liu et al., 2024). This process involves the horizontal transfer of functional mitochondria from healthy donor cells (e.g., mesenchymal stem cells, astrocytes) to stressed or injured recipient cells (e.g., neurons, endothelial cells). In the context of SAE, Kan et al. (2025) demonstrated that astrocyte-to-neuron mitochondrial transfer confers neuroprotection by delivering healthy mitochondria to LPS-stressed neurons, thereby restoring ATP production, reducing oxidative stress, and suppressing apoptosis.

Mechanistically, mitochondrial transfer activates multiple downstream signaling pathways in recipient cells. The incorporation of exogenous functional mitochondria has been shown to reactivate the PGC-1α/NRF2/TFAM biogenesis axis, potentially through improved cellular energetics and reduced ROS-mediated inhibition of this pathway (Liu et al., 2024). Additionally, mitochondrial transfer can enhance mitophagy clearance of remaining damaged mitochondria via the PINK1/Parkin pathway, thereby improving overall mitochondrial quality control (Zhang et al., 2023). The transferred mitochondria also deliver mitochondrial-derived DAMPs at controlled levels, which may precondition recipient cells by mildly activating stress-responsive pathways such as AMP-activated protein kinase (AMPK) and SIRT1, ultimately enhancing cellular resilience (Hu et al., 2019).

Emerging therapeutic strategies are now exploring engineered mitochondrial transplantation using isolated mitochondria or mitochondria-loaded extracellular vesicles. These approaches aim to harness the therapeutic potential of mitochondrial transfer while overcoming limitations such as donor cell availability and immune compatibility. Future research should focus on optimizing delivery methods, elucidating the full spectrum of signaling pathways activated by transferred mitochondria, and evaluating the long-term safety and efficacy of this innovative approach in SAE models.”

In summary, the impairment of mitochondrial biogenesis, mediated through the suppression of the PGC-1α/NRF/TFAM axis, is a critical pathogenic mechanism in SAE that deprives the brain of its metabolic and reparative potential. Therapeutic strategies aimed at reactivating this pathway demonstrate considerable promise in restoring mitochondrial mass and function, thereby offering a powerful approach to preserving neurological function in sepsis.

### 2.3 Mitochondrial oxidative stress

In sepsis, the massive and systemic inflammatory response triggers an overwhelming production of ROS, creating a state of profound oxidative stress that is particularly detrimental to the metabolically active and vulnerable brain. Mitochondria are central players in this process, acting as both a primary source and a critical target of ROS, thereby initiating a vicious cycle of oxidative damage that propagates mitochondrial dysfunction, amplifies neuroinflammation, and drives neuronal death in SAE.

#### 2.3.1 Sources and mechanisms of ROS production

Under physiological conditions, mitochondria produce low levels of ROS, primarily at complexes I and III of the ETC, as byproducts of oxidative phosphorylation. In SAE, this process is drastically accelerated. Systemic inflammation and circulating endotoxins like LPS disrupt the ETC, leading to electron leakage and excessive reduction of electron carriers (Zhao et al., 2024). This results in a significant surge in superoxide anion production. This primary ROS is rapidly converted to hydrogen peroxide and, in the presence of free iron via the Fenton reaction, to the highly reactive hydroxyl radical (•OH) (Zhao et al., 2024). This “oxidative burst” from mitochondria is compounded by extra-mitochondrial sources, such as activated microglia and infiltrating neutrophils that generate ROS via NADPH oxidase enzymes. The integrated result is a veritable storm of ROS that overwhelms the brain’s intrinsic antioxidant defenses (Table 1).

#### 2.3.2 Consequences of oxidative damage

Uncontrolled ROS production inflicts comprehensive damage to critical macromolecules within neurons and glial cells, initiating a cascade of molecular deterioration. The peroxidation of polyunsaturated fatty acids in mitochondrial and cellular membranes compromises membrane integrity and fluidity while generating toxic reactive aldehydes including malondialdehyde (MDA) and 4-hydroxynonenal, which themselves propagate oxidative damage (Kiyuna et al., 2018). Concurrently, ROS induce protein oxidation through amino acid side chain modification and carbonylation, leading to enzymatic dysfunction, misfolding, and aggregation—particularly devastating to electron transport chain components and antioxidant systems, thereby crippling mitochondrial energy production and cellular defense mechanisms (Kiyuna et al., 2018). mtDNA suffers exceptional vulnerability due to its proximity to ROS generation sites and lack of histone protection; oxidative damage to mtDNA results in mutations and deletions that impair synthesis of essential ETC subunits, establishing a vicious cycle of progressive ETC dysfunction and sustained ROS production (Yan et al., 2019).

Beyond direct macromolecular damage, ROS function as pivotal signaling molecules that significantly exacerbate SAE pathology. They potently activate the NLRP3 inflammasome in microglia, thereby bridging oxidative stress to neuroinflammation. Furthermore, ROS promote mitochondrial permeability transition pore (mPTP) opening and serve as core drivers of ferroptosis—an iron-dependent cell death pathway characterized by uncontrolled lipid peroxidation. These coordinated mechanisms underscore how oxidative assault extends beyond simple chemical damage to actively regulate multiple pathological processes in SAE (Zhong et al., 2024) (Table 1).

#### 2.3.3 Endogenous antioxidant defenses

The body employs a sophisticated antioxidant defense system to counter oxidative stress, and enhancing this endogenous system represents a crucial therapeutic approach for SAE. The Nrf2-Kelch-like ECH-associated protein 1(Keap1) axis serves as a master regulator of this defense mechanism (Cui et al., 2021). Under basal conditions, the transcription factor Nrf2 remains bound to its inhibitor Keap1 and undergoes continuous degradation. During oxidative stress or upon specific pharmacological activation, Nrf2 dissociates from Keap1 and translocates to the nucleus, where it initiates the expression of numerous cytoprotective genes (Cui et al., 2021). These include enzymes for glutathione synthesis (GCLC and GCLM), the key anti-ferroptosis enzyme glutathione peroxidase 4 (GPX4), and phase II detoxifying enzymes such as heme oxygenase-. Research has demonstrated that molecular H_2_ and the myokine irisin confer significant neuroprotection in SAE models primarily through activating this Nrf2 pathway, leading to enhanced antioxidant capacity and improved mitochondrial function (Xie et al., 2020; Zhang et al., 2023; Wang et al., 2022).

The AMPK-uncoupling protein 2 (UCP2) axis represents another crucial defense pathway that mitigates oxidative damage through a distinct mechanism (Lei et al., 2021). AMPK, a cellular energy sensor, becomes activated under metabolic stress conditions and subsequently phosphorylates and activates UCP2 (Zhao et al., 2022). Located in the mitochondrial inner membrane, activated UCP2 mildly uncouples oxidative phosphorylation from ATP production, reducing mitochondrial membrane potential and consequently decreasing ROS generation (Zhao et al., 2022). The natural compound *Malvidin* has been shown to protect against SAE by activating this AMPK-*α*/UCP2 axis, directly reducing mitochondrial ROS (mtROS) accumulation, which in turn inhibits NLRP3 inflammasome activation and protects neurons from apoptosis (Zhao et al., 2022) (Table 1).

In summary, the pathogenesis of SAE is critically determined by the dynamic equilibrium between oxidative stress damage and endogenous antioxidant defense systems. Under physiological conditions, a delicate balance exists between mitochondrial ROS production and the scavenging capacity of antioxidant enzymes (e.g., GPX4, catalase, SOD) and non-enzymatic molecules (e.g., glutathione [GSH]). In SAE, this balance is profoundly disrupted: the septic insult triggers an overwhelming burst of mtROS from dysfunctional mitochondria, while simultaneously suppressing key antioxidant defense pathways, particularly the Nrf2-Keap1 and AMPK-UCP2 axes. This imbalance leads to a self-perpetuating cycle of oxidative macromolecular damage (lipid peroxidation, protein carbonylation, mtDNA injury), which further impairs ETC function and exacerbates ROS production. The resulting oxidative stress not only directly injures neurons and glial cells but also serves as a critical signaling mediator that activates the NLRP3 inflammasome, promotes mitochondrial-dependent cell death (apoptosis, pyroptosis, ferroptosis), and disrupts the BBB. Therefore, therapeutic strategies aimed at restoring the oxidative-antioxidative balance, either by reducing mtROS production (e.g., mitochondrial protectants), enhancing endogenous antioxidant capacity (e.g., Nrf2 activators), or both, represent a rational approach to breaking this vicious cycle and preserving neurological function in SAE.

### 2.4 Mitochondria-dependent cell death pathways

Mitochondria are not only the powerhouses of the cell but also central executioners of programmed cell death. In SAE, the convergence of intense bioenergetic stress, oxidative damage, and inflammatory signaling on this organelle triggers the activation of multiple, often interconnected, cell death pathways. The pivotal role of mitochondria in integrating these signals and initiating apoptosis, pyroptosis, and ferroptosis represents a fundamental mechanism underlying the widespread neuronal and glial loss observed in this condition.

#### 2.4.1 Mitochondrial apoptosis: the classic pathway to neuronal demise

The intrinsic, or mitochondrial, apoptotic pathway is a well-established contributor to SAE-associated neuronal death. This pathway is characterized by mitochondrial outer membrane permeabilization (MOMP), a decisive event controlled by the Bcl-2 protein family (Kan et al., 2025). Under the stress of sepsis, pro-apoptotic proteins like Bax and Bak are activated and oligomerize on the outer mitochondrial membrane, forming pores. Concurrently, anti-apoptotic proteins like Bcl-2 are suppressed (Kan et al., 2025). This imbalance leads to MOMP, resulting in the release of several lethal proteins from the mitochondrial intermembrane space into the cytosol. Key among these is cytochrome c, which, once released, forms the “apoptosome” with Apaf-1 and procaspase-9, leading to the activation of the executioner caspase-3 and subsequent systematic cellular dismantlement (Liu et al., 2024; Zhang et al., 2023).

A critical regulator of this process unique to SAE is the mitochondrial serine protease Omi/HtrA2. During septic insult, Omi/HtrA2 translocates from the mitochondrial intermembrane space to the cytosol (Hu et al., 2019; Wang et al., 2018). Once in the cytosol, it exacerbates apoptosis by cleaving and degrading the X-linked inhibitor of apoptosis protein (XIAP), a potent endogenous suppressor of caspase activity. The administration of UCF-101, a specific Omi/HtrA2 inhibitor, has been shown to prevent this cytosolic translocation, attenuate XIAP degradation, suppress caspase-3 activation, and ultimately reduce neuronal apoptosis and improve cognitive outcomes in septic models (Hu et al., 2019; Wang et al., 2018). The critical role of Omi/HtrA2 in SAE-induced apoptosis has been further validated by genetic approaches. Omi/HtrA2 knockout or kinase-dead knockin mice exhibit significantly reduced neuronal apoptosis and improved survival in sepsis models, confirming that the protease activity of Omi/HtrA2 is essential for its pro-apoptotic function (Wang et al., 2018; Wang et al., 2022). Similarly, the inhibition of the purinergic receptor P2X7 receptor (P2X7R) also confers protection by dampening this Omi/HtrA2-mediated apoptotic signaling cascade (Wang et al., 2022) (Table 1).

#### 2.4.2 Mitochondrial regulation of pyroptosis: inflaming cell death

Pyroptosis is a highly inflammatory form of programmed cell death, critically involved in microglial activation and subsequent neuronal injury in SAE. It is primarily executed by GSDMD, which, upon cleavage, forms pores in the plasma membrane, leading to cytokine release and lytic cell death. The cleavage of GSDMD is typically mediated by inflammatory caspases, such as caspase-1, which is itself activated by inflammasome complexes like NLRP3 (Ma et al., 2024; Zhan et al., 2024).

Mitochondria are intimately involved in initiating and amplifying pyroptotic signaling. mtROS serve as a key danger signal for NLRP3 inflammasome activation (Huang et al., 2023). Furthermore, a direct link between mitochondrial dynamics and pyroptosis has been established. The GSDMD/Drp1 signaling pathway illustrates this connection: cleaved GSDMD can promote Drp1-mediated mitochondrial fission, and conversely, mitochondrial fission can facilitate NLRP3 inflammasome assembly, creating a feed-forward loop that amplifies inflammation and cell death (Fu et al., 2024). Another novel mechanism involves OTUD1, a deubiquitinase that exacerbates SAE by promoting the dissociation of hexokinase 2 (HK2) from mitochondria (Jing et al., 2025). This HK2 release triggers microglial pyroptosis via NLRP3 activation, highlighting how mitochondrial metabolic coupling directly regulates this inflammatory death pathway (Jing et al., 2025) (Table 1). Genetic ablation of key pyroptosis mediators has provided definitive evidence for their involvement in SAE. GSDMD knockout mice are protected from LPS-induced cognitive deficits and exhibit reduced hippocampal neuronal loss, accompanied by attenuated Drp1-mediated mitochondrial fission (Fu et al., 2024). Similarly, NLRP3^−^/^−^ mice display markedly reduced microglial pyroptosis, lower IL-1β and IL-18 levels, and preserved synaptic function following septic challenge (Ma et al., 2024).

#### 2.4.3 Mitochondria at the crossroads of ferroptosis: a metabolic catastrophe

Ferroptosis is an iron-dependent form of cell death driven by the catastrophic accumulation of lipid peroxides. Its connection to mitochondria in SAE is profound. Mitochondria are a major site of cellular iron metabolism and ROS generation, both central to ferroptosis execution. In SAE, the delicate balance between lipid peroxidation and its reduction is disrupted. Key defenders against ferroptosis, such as GPX4 and its substrate GSH, are downregulated, while pro-ferroptotic factors like labile iron and lipid peroxidation products (e.g., MDA) increase (Niu et al., 2025).

The central antioxidant transcription factor Nrf2 emerges as a critical suppressor of hippocampal ferroptosis in SAE. Nrf2 activation upregulates genes involved in iron metabolism (e.g., FTH1), GSH synthesis, and antioxidant defense, thereby maintaining mitochondrial lipid membrane integrity. Irisin, an exercise-induced myokine, has been demonstrated to protect against SAE by activating the Nrf2/GPX4 signal axis, thereby inhibiting ferroptosis and preserving neuronal viability (Wang et al., 2022). Similarly, Maresin1 alleviates cognitive impairment by activating the Solute Carrier Family 7 Member 11/GPX4 pathway, which is the core defense system against ferroptosis (Wu et al., 2024). The role of mitochondrial dynamics in this process is further highlighted by findings that Yap1 alleviates ferroptosis partly by inhibiting Drp1-mediated mitochondrial fission, thereby maintaining mitochondrial metabolic homeostasis and reducing lipid peroxidation (Yang et al., 2024) (Table 1). The essential protective role of the Nrf2-GPX4 axis against ferroptosis in SAE has been firmly established through gene knockout models. Nrf2 knockout mice exhibit exacerbated hippocampal ferroptosis, characterized by increased lipid peroxidation, mitochondrial shrinkage, and cognitive decline following CLP-induced sepsis (Duan et al., 2024). Conversely, GPX4 conditional knockout in forebrain neurons recapitulates key features of SAE-associated neurodegeneration, including profound memory impairment and hippocampal neuronal loss, even in the absence of an external septic insult (Wang et al., 2022).

In summary, mitochondria serve as a central platform that coordinates the activation of apoptosis, pyroptosis, and ferroptosis in SAE. These pathways are not isolated but are mechanistically intertwined—mtROS can trigger both pyroptosis and ferroptosis, and Drp1-mediated fission is a common amplifier. The intricate crosstalk between these mitochondria-dependent cell death mechanisms significantly amplifies the initial septic insult, leading to extensive neural damage. Consequently, therapeutic interventions that target these pathways at the mitochondrial level hold immense potential for mitigating brain injury in SAE.

## 3 Neuroinflammation and microglial activation

Neuroinflammation, driven predominantly by the aberrant activation of microglia, the resident immune sentinels of the CNS, constitutes a cornerstone of SAE pathogenesis. In response to peripheral inflammatory signals, microglia undergo a dramatic phenotypic and functional transformation, shifting from a homeostatic surveillance state to a potent pro-inflammatory effector state. This activation initiates a cascade of events that disrupts neuronal function, compromises synaptic integrity, and perpetuates a self-sustaining cycle of inflammation and injury.

### 3.1 Microglial polarization: M1/M2 phenotypes

The initial activation of microglia in SAE is triggered by a multitude of signals, including circulating pathogen-associated molecular patterns (PAMPs, e.g., LPS), DAMPs released from injured cells, and pro-inflammatory cytokines (e.g., TNF-*α*, IL-1β) (Yan et al., 2022). This triggers a spectrum of activation states, broadly categorized into the classical pro-inflammatory M1 phenotype and the alternative anti-inflammatory, pro-repair M2 phenotype. In the acute phase of SAE, the M1 phenotype predominates. These activated microglia upregulate surface markers like CD86 and release a storm of pro-inflammatory mediators, including TNF-α, IL-1β, IL-6, inducible nitric oxide synthase, and cyclooxygenase-2 (COX-2) (Yan et al., 2022). This toxic milieu directly damages neurons, inhibits neurogenesis, and contributes to synaptic pruning and loss. The persistence of this M1-skewed inflammation is a key driver of chronic cognitive impairment. Conversely, the M2 phenotype, associated with the release of anti-inflammatory factors like IL-10 and growth factors, is crucial for resolution of inflammation and tissue repair. The imbalance between M1 and M2 polarization is therefore a critical determinant of SAE outcome (Yan et al., 2022). Pharmacological interventions, such as the Drp1 inhibitor Mdivi-1, have been shown to alleviate cognitive damage not only by improving mitochondrial function but also by shifting microglial polarization from the detrimental M1 state toward the protective M2 state (Hong et al., 2025) (Table 2).

**Table 2:** Neuroinflammation and microglial activation in SAE.

Aspect	Phenotype/Mechanism	Key mediators/pathways	Functional outcomes
Microglial polarization	M1 (Pro-inflammatory)	TNF-α, IL-1β, IL-6, iNOS, COX-2	Neuronal damage, synaptic loss
	M2 (Anti-inflammatory/Repair)	IL-10, growth factors	Resolution, tissue repair
Core amplifier	NLRP3 Inflammasome activation	mtROS, mtDNA, Drp1 → Caspase-1, IL-1β, GSDMD	Pyroptosis, synaptic loss
Other modulators	TRIM45 (↑)	Atg5 → NLRP3 activation	Promotes pyroptosis
	Nogo-A (↑)	SHP-2/NLRP3 imbalance → M1 polarization	ROS ↑, inflammation ↑
	Fgr Kinase (↑)	Inhibits SIRT1/PGC-1α	Worsens mitochondrial function
Protective mechanisms	M2 exosomes (miR-124-3p)	Inhibits ROCK/PTEN pathway	Reduces neuronal apoptosis
	Stanniocalcin-1 (↑)	Suppresses microglial inflammation	Preserves mitochondrial function

This table outlines the roles of microglial polarization, key inflammatory pathways, regulatory modulators, and protective mechanisms in SAE pathogenesis. SAE, Sepsis-associated encephalopathy; TNF-α, Tumor necrosis factor-alpha; IL-1β, Interleukin-1 beta; IL-6, Interleukin-6; iNOS, Inducible nitric oxide synthase; COX-2, Cyclooxygenase-2; IL-10, Interleukin-10; mtROS, Mitochondrial reactive oxygen species; mtDNA, Mitochondrial DNA; TRIM45, Tripartite motif-containing protein 45; Atg5, Autophagy related 5; Nogo-A, Neurite outgrowth inhibitor A; SHP-2, Src homology region 2-containing protein tyrosine phosphatase-2; Fgr, Gardner-Rasheed feline sarcoma viral oncogene homolog; SIRT1, Sirtuin 1; PGC-1α, Peroxisome proliferator-activated receptor-gamma coactivator-1α; ROCK1/2, Rho-associated coiled-coil containing protein kinase 1/2; PTEN, Phosphatase and tensin homolog; Akt, Protein kinase B; mTOR, Mechanistic target of rapamycin.

### 3.2 The NLRP3 inflammasome as an inflammation amplifier

The NLRP3 inflammasome is a multi-protein complex that serves as a critical intracellular platform for amplifying the inflammatory response in SAE (Luo et al., 2025). Its activation within microglia is a two-step process: priming (initiated by signals like LPS that upregulate NLRP3 and pro-IL-1β) and activation (triggered by a second signal such as extracellular ATP or mitochondrial DAMPs). Once assembled, the NLRP3 inflammasome activates caspase-1, which then cleaves pro-IL-1β and pro-IL-18 into their active, secreted forms and cleaves GSDMD to execute pyroptosis (Luo et al., 2025).

Mitochondrial dysfunction is a key trigger for NLRP3 activation. mtROS, released from damaged mitochondria, and mtDNA, when released into the cytosol, are potent DAMPs that directly promote NLRP3 inflammasome assembly (Moraes et al., 2023). Furthermore, the process of mitochondrial fission, mediated by Drp1, facilitates NLRP3 activation, creating a pathogenic link between mitochondrial dynamics and inflammation (Fu et al., 2024). The consequences are severe: NLRP3-driven release of IL-1β induces excitatory synaptic loss, either directly or through the release of IL-1β-enriched microvesicles from microglia, which have been shown to suppress neurite outgrowth and damage synapses (Moraes et al., 2023). The significance of this pathway is underscored by studies showing that inhibition of NLRP3 (e.g., with MCC950) or its upstream regulators (e.g., mtROS) effectively reduces neuroinflammation and cognitive deficits in SAE models (Xie et al., 2020) (Table 2).

### 3.3 Additional signaling pathways and modulators

Beyond the NLRP3 inflammasome, microglial-mediated neuroinflammation in SAE is finely regulated by a network of additional signaling molecules and pathways. The E3 ubiquitin ligase TRIM45 exacerbates disease progression by facilitating NLRP3 inflammasome activation through its regulation of Autophagy Related 5 expression, thereby disrupting autophagic flux and promoting microglial pyroptosis (Huang et al., 2023). Genetic knockdown of TRIM45 has been demonstrated to reduce neuronal damage and improve cognitive outcomes (Huang et al., 2023). Similarly, Nogo-A, traditionally recognized for its role in inhibiting neurite outgrowth, contributes to SAE pathogenesis by modulating the SHP-2/NLRP3 balance in microglia. This modulation promotes polarization toward the pro-inflammatory M1 phenotype and induces ROS production, thereby amplifying the inflammatory cascade (Liu et al., 2024).

In the realm of kinase signaling, inhibition of Fgr, a member of the Src family kinases, attenuates SAE by ameliorating both mitochondrial dysfunction and neuroinflammation through the SIRT1/PGC-1α signaling pathway (Liu et al., 2023). This highlights the potential of targeting upstream kinases to modulate the inflammatory response. Furthermore, metabolic reprogramming represents another crucial regulatory layer, wherein activated microglia undergo a Warburg-like effect, shifting toward glycolysis to meet their heightened energetic and biosynthetic demands. Interestingly, the sedative agent propofol has been found to inhibit this LPS-induced metabolic reprogramming by suppressing the ROS/PI3K/Akt/mTOR/HIF-1α axis, thereby exerting anti-inflammatory effects (Guan et al., 2023) (Table 2).

Collectively, these diverse signaling pathways and modulators work in concert to fine-tune microglial activation and neuroinflammatory responses, offering multiple potential intervention points for SAE treatment beyond the canonical inflammasome pathway.

### 3.4 Protective and resolving mechanisms

Not all microglial responses are detrimental. The M2 phenotype and its derivatives play a vital role in limiting damage. A key mechanism of protection is through the release of exosomes. M2 microglia-derived exosomes have been shown to carry miR-124-3p, which, upon delivery to neurons, targets and inhibits ROCK1 and ROCK2. This action attenuates the ROCK/PTEN/Akt/mTOR signaling pathway, thereby reducing glutamate-induced neuronal apoptosis and conferring neuroprotection (Zhu et al., 2024). This represents a sophisticated form of intercellular communication whereby microglia can exert a paracrine protective effect. Additionally, proteins like Stanniocalcin-1 inhibit the pro-inflammatory response in microglia and protect mitochondrial function, offering significant neuroprotection against SAE (Bonfante et al., 2021) (Table 2).

In conclusion, microglial activation is a central orchestrator of SAE pathology. The transition to a pro-inflammatory state, powerfully amplified by the NLRP3 inflammasome and modulated by a network of signaling pathways and metabolic shifts, creates a hostile CNS environment that drives neuronal dysfunction and death. Understanding the nuanced interplay between these mechanisms, including the potential for beneficial microglial functions, is essential for developing targeted therapies that can suppress detrimental neuroinflammation while preserving the innate repair capabilities of the brain.

## 4 BBB disruption

The BBB is a highly specialized and dynamic interface that rigorously regulates the exchange of molecules between the circulatory system and the CNS, thereby maintaining the delicate microenvironment essential for normal neuronal function. Its integrity is paramount for brain homeostasis, and its disruption is a hallmark pathological event in SAE, facilitating the unregulated entry of neurotoxic blood-derived substances, inflammatory cells, and cytokines into the brain parenchyma, which exacerbates neuroinflammation and directly inflicts neuronal injury.

### 4.1 Anatomical and functional foundations of the BBB

The BBB is not a passive barrier but a neurovascular unit composed of specialized endothelial cells, pericytes, astrocyte end-feet, and microglia. The brain microvascular endothelial cells (BMECs) are the core structural component, characterized by continuous TJs and adherens junctions that seal the paracellular pathway (Tang et al., 2025). Key TJ proteins include claudin-5, occludin, and zonula occludens-1 (ZO-1), which are critical for maintaining high transendothelial electrical resistance and low permeability. Furthermore, BMECs exhibit low rates of transcytosis and express a battery of specific transport systems and efflux pumps (e.g., P-glycoprotein), which selectively control the transcellular passage of nutrients and actively exclude toxins (Tang et al., 2025) (Table 3). The breakdown of the BBB during sepsis is a multifactorial process driven by the systemic inflammatory response, which directly targets each component of the neurovascular unit.

**Table 3:** Mechanisms of blood–brain barrier disruption in SAE.

Target/Mechanism	Key factors/pathways	Impact on BBB	Experimental interventions
Inflammatory mediators	Poldip2/NF-κB/COX-2 axis	Vascular hyperpermeability	Poldip2 knockdown, Meloxicam
Endothelial mitochondrial dysfunction	Drp1-mediated fission → ROS ↑	TJ damage, bioenergetic failure	Mdivi-1, P110
	Omi/HtrA2 → XIAP ↓ → Caspase-3 activation	Apoptosis of BMECs	UCF-101
TJ disassembly	Phosphorylation/internalization of Claudin-5, Occludin, ZO-1	Paracellular leakage	–
	S100B/RAGE/Ceramide → Drp1 activation	Mitochondrial fission → TJ loss	–
Impairment of transport	P-glycoprotein dysfunction	Reduced toxin efflux	–
Pericytes and astrocytes	Pericyte detachment, astrocyte swelling → S100B, MMPs ↑	BBB destabilization, degradation of basal lamina	H_2_-rich saline (↓GFAP, MMP-9)

This table details the mechanisms contributing to BBB dysfunction in SAE, including inflammatory signaling, endothelial mitochondrial dysfunction, tight junction disassembly, transport impairment, and the roles of pericytes and astrocytes. SAE, Sepsis-associated encephalopathy; BBB, Blood–brain barrier; Poldip2, Polymerase delta-interacting protein 2; NF-κB, Nuclear factor kappa B; COX-2, Cyclooxygenase-2; BMECs, Brain microvascular endothelial cells; Drp1, Dynamin-related protein 1; ROS, Reactive oxygen species; TJ, Tight junction; Omi/HtrA2, Serine protease Omi/mitochondrial; XIAP, X-linked inhibitor of apoptosis protein; ZO-1, Zonula occludens-1; S100B, S100 calcium-binding protein B; RAGE, Receptor for advanced glycation endproducts; GFAP, Glial fibrillary acidic protein; MMP-9, Matrix metalloproteinase-9.

#### 4.1.1 Inflammatory mediator-induced dysfunction

Peripheral inflammatory mediators, particularly LPS and cytokines like TNF-*α* and IL-1β, can directly or indirectly compromise BBB integrity. A key mechanism involves the activation of the transcription factor NF-κB within BMECs (Huang et al., 2023). This activation leads to the upregulation of pro-inflammatory genes, including COX-2. The subsequent production of prostaglandin E2 is a potent inducer of vascular permeability. The protein Poldip2 has been identified as a novel regulator of this pathway. Silencing Poldip2 *in vitro* blocked LPS-induced p65 nuclear translocation, COX-2 expression, and endothelial hyperpermeability. *In vivo*, heterozygous deletion of Poldip2 conferred protection against LPS-induced BBB disruption, an effect that was mimicked by the COX-2 inhibitor meloxicam, underscoring the significance of the Poldip2/NF-κB/COX-2 axis in SAE-related BBB damage (Kikuchi et al., 2019) (Table 3).

#### 4.1.2 Endothelial mitochondrial dysfunction

BMECs possess an exceptionally abundant population of mitochondria, which provide crucial ATP support for maintaining TJ integrity and active transport processes. Consequently, mitochondrial dysfunction emerges as a central driver of BBB disruption in SAE. Mirroring the pathological events observed in neurons, excessive mitochondrial fission represents a key pathological mechanism in BMECs. Activation of Drp1 triggers mitochondrial fragmentation, leading to increased ROS production and bioenergetic failure (Haileselassie et al., 2020; Wang et al., 2024). This oxidative stress directly damages TJs proteins and activates signaling pathways that promote their internalization and degradation. Both the Drp1 inhibitor *Mdivi-1* and the specific peptide inhibitor *P110*, which disrupts the Drp1-Fis1 interaction, have demonstrated efficacy in attenuating BBB leakage, preserving claudin-5 and occludin expression, and improving outcomes in septic models, thereby highlighting the therapeutic potential of stabilizing the mitochondrial network in endothelial cells (Haileselassie et al., 2020; Wang et al., 2024).

Concurrently, the mitochondrial-dependent apoptotic pathway becomes potently activated in BMECs during sepsis. The serine protease Omi/HtrA2 plays a critical role in this process (Hu et al., 2019; Wang et al., 2018). Upon activation, Omi/HtrA2 translocates from mitochondria to the cytosol, where it promotes apoptosis through degradation of XIAP, leading to subsequent caspase-3 activation. This apoptotic cell death directly compromises the endothelial barrier integrity. Experimental evidence shows that pharmacological inhibition of Omi/HtrA2 with *UCF-101* or its genetic knockdown significantly reduces LPS-induced BMEC apoptosis, preserves TJ protein expression, and improves BBB integrity in both *in vitro* and *in vivo* settings (Hu et al., 2019; Wang et al., 2018) (Table 3).

These findings collectively demonstrate that targeted intervention in mitochondrial fission and apoptotic pathways may represent an effective strategy for preserving BBB function in SAE.

#### 4.1.3 TJ disassembly

The inflammatory milieu of SAE directly targets TJ complexes. Pro-inflammatory cytokines and ROS can activate various kinases that phosphorylate TJ proteins, leading to their endocytosis and degradation (Zhang et al., 2021). This disassembly of claudin-5, occludin, and ZO-1 opens the paracellular route, allowing for the unregulated passage of substances. This is evidenced by increased extravasation of Evans blue dye and FITC-dextran in animal models of SAE. The S100B/RAGE/ceramide pathway has been implicated in this process, where elevated S100B engages its receptor RAGE, leading to ceramide accumulation, which in turn promotes Drp1-mediated mitochondrial fission and subsequent TJ loss, forming a vicious cycle of damage (Zhang et al., 2021) (Table 3).

#### 4.1.4 Transcellular pathway and efflux system impairment

In addition to the paracellular route, the transcellular pathway may also be enhanced through increased vesicular transport (transcytosis). Moreover, the function of critical efflux pumps like P-glycoprotein can be impaired during inflammation, reducing the brain’s ability to export toxins and drugs, further compounding neuronal exposure to harmful substances (Bourguinat et al., 2010) (Table 3).

#### 4.1.5 Contributions of pericytes and astrocytes

Pericytes, which provide structural support and regulate capillary blood flow, can detach from capillaries in sepsis, further destabilizing the BBB. Astrocytic end-feet surrounding the vasculature become swollen and reactive, releasing additional inflammatory factors like S100B and MMPs, which directly degrade TJ proteins and the basal lamina. H_2_-rich saline has been shown to attenuate BBB damage partly by reducing GFAP (an astrocyte marker) immunoreactivity and MMP-9 activity (Dumbuya et al., 2023) (Table 3).

In conclusion, BBB disruption in SAE is a complex process initiated by systemic inflammation and executed through multiple converging mechanisms: inflammatory signaling activation, profound mitochondrial dysfunction in endothelial cells, disassembly of TJs, and dysfunction of supporting cells. The central role of mitochondrial fission and apoptosis in BMECs provides a compelling therapeutic rationale for targeting mitochondrial stability to preserve this critical barrier and mitigate the entry of neurotoxic substances into the brain during sepsis.

## 5 The gut-brain axis and mitochondrial metabolomics

The gut-brain axis, a complex, bidirectional communication network linking the gastrointestinal tract with the central nervous system, has emerged as a critical modulator of systemic and neuro-inflammation in sepsis. Growing evidence indicates that sepsis-induced dysbiosis, a disruption of the gut microbial ecosystem, plays a pivotal role in the pathogenesis of SAE, primarily through mechanisms that converge on mitochondrial function within the brain. The integration of metabolomics, the comprehensive study of small-molecule metabolites, is providing unprecedented insights into how gut-derived signals drive mitochondrial dysfunction and neuronal injury.

### 5.1 Impact of gut dysbiosis on brain function

Sepsis triggers a profound alteration in the composition and diversity of the gut microbiota, characterized by a loss of beneficial commensal bacteria and an expansion of pathobionts. This dysbiotic state is not merely a consequence of critical illness but actively contributes to SAE progression. The compromised gut barrier, a phenomenon often termed “leaky gut,” allows for the translocation of bacteria and their products (e.g., LPS) into the systemic circulation, perpetuating a state of chronic inflammation and potentially amplifying the initial septic insult. A seminal study by Huang et al. (2026) demonstrated the causal role of the gut microbiota in SAE. They showed that fecal microbiota transplantation (FMT) from healthy donors to LPS-treated rats preserved gut microbial structure, improved intestinal barrier integrity (as evidenced by maintained ileal villus length), and increased systemic levels of short-chain fatty acids (SCFAs). Crucially, this restoration of gut health was associated with a remarkable mitigation of sensorimotor deficits and a protection of mitochondrial bioenergetics in cortical astrocytes. Conversely, administration of feces from endotoxemic donors exacerbated these detrimental effects, underscoring the pivotal role of gut microbial status in determining SAE outcomes (Table 4).

**Table 4:** The role of the gut-brain axis and cerebral metabolomics in SAE.

Factor	Key alterations	Impact on brain and mitochondria	Experimental evidence/intervention
Gut dysbiosis	↓Commensals, ↑pathobionts, leaky gut	LPS translocation → neuroinflammation ↑	FMT from healthy donors → preserves barrier, ↑SCFAs, improves mitochondrial function
Microbial metabolites	↓SCFAs (butyrate, acetate, propionate)	Loss of anti-inflammatory signaling	FMT ↑SCFAs → protects astrocytes
Cerebral metabolomics	↓GSH, ↓GSH/GSSG ratio	Oxidative stress → mPTP opening → cell death	CypD knockout maintains GSH, improves survival
Mitochondrial transfer	Impaired astrocyte-to-neuron transfer	Loss of metabolic support to neurons	Astrocyte-conditioned medium → attenuates neuronal damage

This table summarizes the impact of gut dysbiosis, microbial metabolites, cerebral metabolomic changes, and intercellular mitochondrial transfer on brain mitochondrial function and SAE pathology. SAE, Sepsis-associated encephalopathy; LPS, Lipopolysaccharide; FMT, Fecal microbiota transplantation; SCFAs, Short-chain fatty acids; NF-κB, Nuclear factor kappa B; GSH, Glutathione; GSSG, Glutathione disulfide; mPTP, Mitochondrial permeability transition pore; CypD, Cyclophilin D.

Notably, specific bacterial families and genera have been implicated in modulating brain function through the gut-brain axis. Beneficial commensals, particularly those producing SCFAs such as *Lactobacillus, Bifidobacterium, Faecalibacterium* (e.g., *F. prausnitzii*), and *Roseburia*, are associated with anti-inflammatory and neuroprotective effects (Huang et al., 2026; Kobayashi et al., 2022). In contrast, sepsis-induced dysbiosis is characterized by an expansion of potentially pathogenic taxa, including *Escherichia, Shigella,* and *Enterococcus*, which may exacerbate systemic inflammation and neuroinflammation through the release of endotoxins and pro-inflammatory metabolites (Dumbuya et al., 2022). The relative abundance of these microbial populations critically influences the production of neuroactive metabolites (e.g., SCFAs, tryptophan metabolites) and the integrity of the intestinal barrier, thereby modulating the severity of SAE.

### 5.2 Microbial metabolites as key messengers

Gut microbes produce a vast repertoire of metabolites that can enter the bloodstream, cross the BBB, and directly influence brain function. Among these, SCFAs, such as acetate, propionate, and butyrate, produced by the fermentation of dietary fiber, are of particular importance. SCFAs are not merely metabolic byproducts; they are potent signaling molecules with anti-inflammatory and neuroprotective properties. They can inhibit histone deacetylases and activate specific G-protein-coupled receptors (e.g., GPR41, GPR43, GPR109a), leading to the suppression of NF-κB signaling and a reduction in the production of pro-inflammatory cytokines. The study by Huang et al. (2026) directly linked the increased SCFA levels following healthy FMT to enhanced anti-inflammatory cytokine release and the preservation of mitochondrial function in astrocytes, positioning SCFAs as critical mediators linking a healthy gut to cerebral bioenergetic health (Table 4).

### 5.3 Cerebral metabolomic signatures in SAE

Metabolomic profiling of brain tissue offers a powerful, unbiased approach to identify the global metabolic disturbances that underpin SAE. Research by Kobayashi et al. (2022) utilizing cyclophilin D (CypD) knockout mice provided profound insights. CypD is a key regulator of the mPTP, and its deletion conferred protection in a CLP model of sepsis. Metabolomic analysis of brain tissue revealed that the primary protective mechanism was the maintenance of reduced GSH levels and a high GSH/GSSG (oxidized glutathione) ratio in knockout animals following sepsis (Kobayashi et al., 2022). The critical role of mPTP opening in SAE pathogenesis was elegantly demonstrated using CypD knockout mice (Kobayashi et al., 2022). CypD is a key regulatory component of the mPTP, and its genetic deletion confers remarkable protection in a CLP model of sepsis. Metabolomic analysis revealed that CypD^−/−^ mice maintained significantly higher levels of reduced GSH and a preserved GSH/GSSG ratio in brain tissue following sepsis, indicating enhanced mitochondrial redox capacity and resistance to oxidative stress-induced mPTP opening (Kobayashi et al., 2022). These findings highlight that the resilience of the mitochondrial antioxidant system, crucially dependent on GSH, is a decisive factor in surviving septic encephalopathy. The inability to maintain this redox balance in wild-type mice leads to oxidative damage, mPTP opening, and amplified cell death (Table 4).

### 5.4 Intercellular mitochondrial transfer

A groundbreaking discovery in neuro-glial communication is the transfer of whole mitochondria from astrocytes to neurons, a process that may represent a fundamental mechanism of metabolic support under stress. Kan et al. (2025) investigated this phenomenon in an *in vitro* model of SAE. They found that when neurons were exposed to LPS, mitochondria from healthy astrocytes migrated toward and connected with the stressed neurons. The administration of astrocyte-conditioned mediu, which contained these transferred mitochondria or mitochondrial components, significantly attenuated LPS-induced alterations in proteins related to apoptosis and mitochondrial dynamics in neurons (Kan et al., 2025). RNA sequencing and lipidomic analysis further revealed that this mitochondrial transfer induced significant changes in the neuronal transcriptome and lipid profile, notably increasing phosphatidylserine levels, which are implicated in anti-inflammatory signaling. This suggests that astrocyte-neuron mitochondrial transfer may protect against SAE by providing functional mitochondria and modifying the neuronal metabolic and inflammatory state (Table 4).

In conclusion, the gut-brain axis and cerebral metabolomic landscape are integral to the pathophysiology of SAE. Sepsis-induced gut dysbiosis impairs the production of beneficial metabolites like SCFAs and promotes systemic inflammation, which directly and indirectly damages cerebral mitochondria. Meanwhile, metabolomic studies reveal that the failure of mitochondrial antioxidant systems is a critical event in SAE. The emerging phenomenon of intercellular mitochondrial transfer adds a new layer of complexity, suggesting that the brain has intrinsic, cell-to-cell support mechanisms that, if therapeutically harnessed, could ameliorate bioenergetic failure. Together, these insights advocate for a holistic view of SAE that incorporates gut health and systemic metabolism, opening avenues for novel therapies such as probiotics, prebiotics, or metabolites designed to preserve mitochondrial function in the brain.

## 6 Therapeutic strategies targeting mitochondria and neuroinflammation

The intricate interplay between mitochondrial dysfunction and neuroinflammation in SAE provides a rich landscape for therapeutic intervention. Targeting these interconnected pathways offers a promising approach to ameliorate brain injury and improve neurological outcomes. Based on recent preclinical evidence, several strategic approaches have emerged, ranging from direct mitochondrial protection to modulation of inflammatory signaling and metabolic support.

### 6.1 Medical gasses and metabolic modulators

#### 6.1.1 Molecular H_2_

Molecular H_2_ has garnered significant attention for its pleiotropic protective effects in SAE, attributable to its unique biochemical properties. It exhibits a selective capacity to neutralize cytotoxic ROS, particularly the hydroxyl radical (•OH), while its small molecular size and high diffusibility enable it to readily penetrate cellular membranes and organelles, including mitochondria.

**Figure 1:**
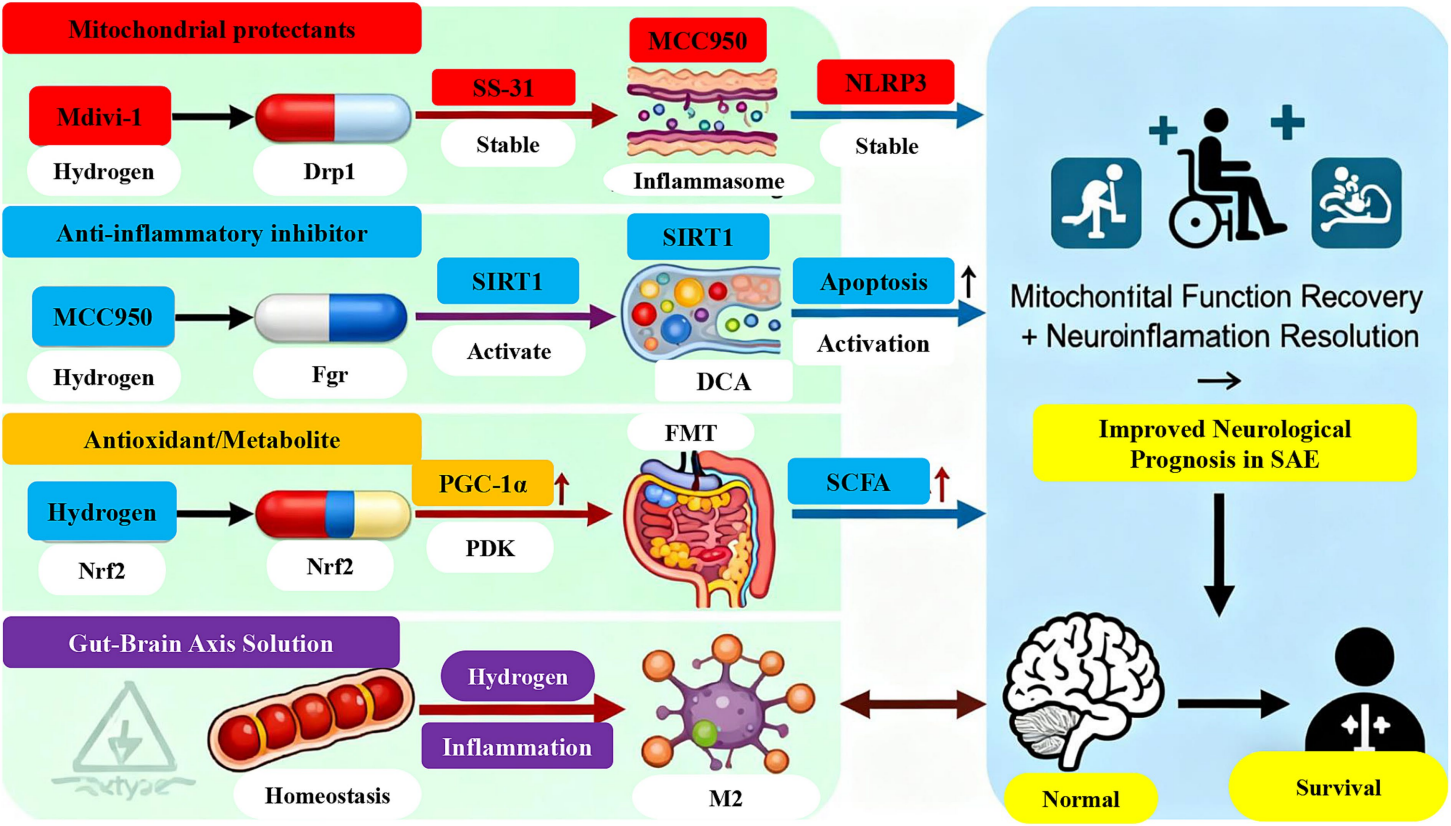
Therapeutic strategies targeting the mitochondrial-neuroinflammatory axis in sepsis-associated encephalopathy (SAE). The left panel categorizes representative interventions into four classes based on their primary targets: mitochondrial protectants, anti-inflammatory inhibitors, antioxidant/metabolic modulators, and gut–brain axis regulators. The right panel summarizes key therapeutic outcomes in SAE models, including restored mitochondrial function (elevated ATP, reduced ROS, recovered membrane potential), resolved neuroinflammation (lower pro-inflammatory cytokines, microglial shift from M1 to M2), and improved neurological outcomes (reduced apoptosis, preserved synapses, enhanced cognition, and survival). Together, these strategies disrupt the pathogenic cycle of mitochondrial dysfunction and neuroinflammation, offering promising therapeutic avenues for SAE.

The therapeutic benefits of H_2_ are mediated through multiple interconnected pathways that converge on mitochondrial and inflammatory homeostasis. Primarily, H_2_ exerts potent antioxidant and anti-apoptotic effects by mitigating mitochondrial oxidative stress, thereby preserving MMP, preventing cytochrome c release, and inhibiting caspase-3-dependent apoptosis. In parallel, H_2_ displays broad anti-inflammatory properties; whether administered via inhalation or as H_2_-rich saline, it significantly suppresses microglial activation and the release of pro-inflammatory cytokines such as TNF-*α*, IL-1β, and IL-6. A key mechanism underlying this immunomodulation involves the upregulation of the transcription factor Nrf2, which potently inhibits the NLRP3 inflammasome pathway, thereby reducing the production of IL-1β and IL-18 (Xie et al., 2020; Dumbuya et al., 2023). Furthermore, H_2_ supports cellular bioenergetics by activating the PGC-1α/NRF2/TFAM axis, thereby enhancing mitochondrial biogenesis, as evidenced by increased ATP levels, restored complex I activity, and improved cognitive function in septic mice (Cui et al., 2024; Xie et al., 2021). Beyond biogenesis, H_2_ also contributes to mitochondrial quality control by promoting the expression of mitophagy-related proteins (PINK1, PARKIN) and fusion protein MFN2, while concurrently suppressing the mitochondrial fission protein Drp1 (Zhang et al., 2023). Therapeutically, both H_2_ gas inhalation (e.g., 2–67%) and H_2_-rich saline injections have demonstrated significant efficacy across various SAE models, consistently improving survival rates, reducing neuronal damage, and rescuing cognitive deficits (Cui et al., 2024; Zhang et al., 2023; Dumbuya et al., 2022) (Figure 1; Table 5).

**Table 5:** Representative therapeutic strategies targeting the mitochondrial-neuroinflammatory axis in SAE.

Therapeutic category	Representative agent	Primary target/mechanism	Key outcomes	Evidence in SAE models
Medical gasses and metabolic modulators	Molecular H_2_	Nrf2 activation, NLRP3 inhibition, PGC-1α ↑	Antioxidant, anti-inflammatory, improves bioenergetics	Murine CLP/LPS
	Dichloroacetate (DCA)	PDK inhibition → oxidative phosphorylation ↑, Drp1 ↓	Improves mitochondrial function, reduces neuroinflammation	Rat SAE model
Mitochondria-targeted agents	SS-31 (Elamipretide)	Binds cardiolipin, stabilizes cristae, inhibits mPTP	Reverses mitochondrial dysfunction	Murine CLP
	Mdivi-1	Drp1 inhibition → fission ↓, M2 polarization ↑	Improves cognition, reduces inflammation	Murine LPS/CLP
Natural compounds	(-)-Epicatechin	AMPK activation → anti-inflammatory, mitochondrial protection	Improves memory, synaptic plasticity	Rat SAE model
	Malvidin	AMPK-α/UCP2 → mtROS ↓, NLRP3 inhibition	Reduces apoptosis, inflammation	Murine SAE model
	STS	SIRT1/PGC-1α → biogenesis ↑	Reduces neuroinflammation, neuronal injury	Murine SAE model
Specific inhibitors	Fgr kinase inhibitor	SIRT1/PGC-1α activation → mitochondrial and inflammatory improvement	Attenuates SAE pathology	Murine CLP model
	MCC950	NLRP3 inflammasome inhibition	Reduces IL-1β, neuroinflammation	Murine SAE model
	P2X7R inhibitor	NLRP3 ↓, Omi/HtrA2 pathway inhibition	Improves survival, cognition	Murine SAE model
Gut-brain axis modulation	FMT	Restores microbiota, ↑SCFAs, ↓inflammation	Improves sensorimotor function, mitochondrial bioenergetics	Rat LPS model

This table categorizes promising therapeutic interventions for SAE based on preclinical evidence, listing representative agents, their primary targets or mechanisms, and the experimental models in which efficacy has been demonstrated. SAE, Sepsis-associated encephalopathy; H_2_, Molecular hydrogen; Nrf2, Nuclear factor erythroid 2–related factor 2; NLRP3, NOD-, LRR- and pyrin domain-containing protein 3; PGC-1α, Peroxisome proliferator-activated receptor-gamma coactivator-1α; CLP, Cecal ligation and puncture; LPS, Lipopolysaccharide; DCA, Dichloroacetate; PDK, Pyruvate dehydrogenase kinase; Drp1, Dynamin-related protein 1; mPTP, Mitochondrial permeability transition pore; AMPK, AMP-activated protein kinase; UCP2, Uncoupling protein 2; mtROS, Mitochondrial reactive oxygen species; STS, Sodium Tanshinone IIA Sulfonate; SIRT1, Sirtuin 1; P2X7R, P2X purinoceptor 7; Omi/HtrA2, Serine protease Omi/mitochondrial; FMT, Fecal microbiota transplantation; SCFAs, Short-chain fatty acids.

#### 6.1.2 Dichloroacetate (DCA)

DCA, a small-molecule metabolic modulator, exerts its protective effects in SAE primarily by inhibiting pyruvate dehydrogenase kinase. This action promotes the activity of pyruvate dehydrogenase, thereby shifting cellular metabolism from glycolysis toward glucose oxidation and enhancing mitochondrial oxidative phosphorylation. By facilitating this metabolic reprogramming, DCA significantly ameliorates mitochondrial dysfunction under septic conditions.

In a rat model of SAE, treatment with DCA (25–100 mg/kg) was shown to exert multi-faceted benefits (Wang et al., 2024). It effectively suppressed mitochondrial fission by downregulating the expression of Drp1 and its phosphorylated form. Concurrently, DCA attenuated neuroinflammation, as evidenced by reduced activation of astrocytes and microglia along with decreased levels of cerebral inflammatory cytokines. Furthermore, DCA treatment enhanced bioenergetic capacity, leading to elevated ATP levels, improved mitochondrial complex I activity, and an increased NAD^+^/NADH ratio. These coordinated improvements in mitochondrial dynamics, inflammatory status, and energy metabolism collectively contributed to the observed recovery of neurological and cognitive function (Wang et al., 2024) (Figure 1; Table 5).

### 6.2 Mitochondria-targeted and protective agents

#### 6.2.1 SS-31 (Elamipretide)

SS-31 represents a pioneering mitochondria-targeted peptide that selectively accumulates in the inner mitochondrial membrane. Its mechanism of action centers on the specific binding to cardiolipin, a phospholipid crucial for maintaining cristae structure and mitochondrial function. By stabilizing cardiolipin, SS-31 preserves cristae integrity, thereby preventing the opening of the mPTP and subsequent cytochrome c release (Wu et al., 2015). Furthermore, this interaction enhances the mitochondrial antioxidant capacity through ROS scavenging and improves electron transport chain efficiency. As a result, SS-31 effectively reverses mitochondrial dysfunction, suppresses neuronal apoptosis and neuroinflammation, and ultimately ameliorates cognitive deficits and reduces mortality in CLP-induced SAE mice (Wu et al., 2015) (Figure 1; Table 5).

#### 6.2.2 Mdivi-1

As a selective inhibitor of the mitochondrial fission protein Drp1, *Mdivi-1* directly counteracts the aberrant mitochondrial dynamics observed in SAE (Dai et al., 2025; Hong et al., 2025). By specifically inhibiting Drp1 GTPase activity, *Mdivi-1* prevents excessive mitochondrial fragmentation and promotes the restoration of a fused, functional mitochondrial network. This structural stabilization facilitates the recovery of bioenergetic function, as evidenced by increased ATP production and attenuated oxidative stress. Furthermore, the improvement in mitochondrial integrity extends to immunomodulatory effects: *Mdivi-1* attenuates neuroinflammation by shifting microglial polarization from the pro-inflammatory M1 phenotype toward the anti-inflammatory M2 phenotype. This phenotypic transition is associated with suppressed pyroptosis and enhanced autophagosome formation, collectively contributing to neuroprotection in experimental SAE models (Dai et al., 2025; Hong et al., 2025) (Figure 1; Table 5).

### 6.3 Natural compounds and phytochemicals

A multitude of plant-derived compounds demonstrate potent multi-target effects in SAE by concurrently addressing mitochondrial dysfunction and neuroinflammation. (-)-*Epicatechin*, a dietary flavonoid, improves memory and behavioral performance in SAE models through ameliorating neuroinflammation, preserving mitochondrial function, and enhancing synaptic plasticity, potentially via activation of the AMPK signaling pathway (Ling et al., 2022). *Capsaicin*, the active component of chili peppers, attenuates SAE pathology by simultaneously inhibiting neuroinflammation and apoptosis while activating BNIP3/NIX-mediated mitophagy, a selective autophagy pathway responsible for clearing damaged mitochondria (Zhang et al., 2025). Similarly, *fisetin* ameliorates cognitive impairment by promoting mitophagy and suppressing NLRP3 inflammasome activation in cerebral microvascular endothelial cells, thereby reducing IL-1β secretion into the CNS (Ding et al., 2022). *Malvidin* exerts its protective effects through activation of the AMPK-*α*/UCP2 axis, resulting in reduced mtROS accumulation, inhibition of NLRP3 inflammasome activation, and protection against neuronal apoptosis (Zhao et al., 2022). STS, derived from the traditional Chinese herb Danshen, alleviates neuroinflammation-induced hippocampal neuronal injury by activating the SIRT1/PGC-1α/NRF1/TFAM pathway, thereby enhancing mitochondrial biogenesis and function (Song et al., 2026). Furthermore, polydatin, a resveratrol glucoside, mitigates SAE by activating Sirt1, which subsequently inhibits p38 phosphorylation, leading to reduced neuroinflammation and mitochondrial protection (Huang et al., 2022). These natural compounds collectively represent promising multi-target therapeutic strategies for SAE intervention through their coordinated effects on mitochondrial homeostasis and neuroinflammatory pathways (Figure 1; Table 5).

### 6.4 Specific pharmacological inhibitors

Targeting specific nodes within the inflammatory and cell death signaling networks represents a promising therapeutic strategy for SAE. Inhibition of Fgr, a Src family tyrosine kinase upregulated in SAE, attenuates neuroinflammation, oxidative stress, and mitochondrial dysfunction through activation of the SIRT1/PGC-1α signaling pathway (Liu et al., 2023). Similarly, pharmacological blockade of the P2X7R, a key mediator of NLRP3 inflammasome activation, improves survival and cognitive function by reducing ROS production and suppressing the Omi/HtrA2 apoptotic pathway (Wang et al., 2022). Direct targeting of the NLRP3 inflammasome itself with compounds such as MCC950 effectively suppresses caspase-1 activation, IL-1β release, and subsequent neuroinflammation and neuronal apoptosis (Xie et al., 2020). Additionally, the chemical chaperone 4-phenylbutyrate addresses endoplasmic reticulum stress—closely linked to mitochondrial function—and alleviates cognitive decline and neuroinflammation via the IRE1α/Xbp1s-Ca^2+^ pathway, thereby restoring mitochondrial calcium homeostasis (Xiong et al., 2024). Together, these targeted approaches demonstrate the therapeutic potential of precision intervention in specific pathogenic pathways of SAE (Figure 1; Table 5).

### 6.5 Modulation of the gut-brain axis

Modulation of the gut-brain axis has emerged as a novel therapeutic approach for SAE, with FMT demonstrating promise. Experimental studies show that transplanting gut microbiota from healthy donors to septic rats preserves intestinal barrier integrity and enhances production of beneficial SCFAs (Huang et al., 2026). These changes subsequently reduce systemic and cerebral inflammation while restoring mitochondrial bioenergetics in cortical astrocytes. Through these coordinated mechanisms, FMT effectively mitigates sepsis-induced sensorimotor impairment (Huang et al., 2026). These findings collectively demonstrate the therapeutic potential of targeting the gut microbiome to indirectly support brain mitochondrial function and neural homeostasis in SAE (Figure 1; Table 5).

In summary, the therapeutic landscape for SAE is rapidly evolving toward multi-target strategies that concurrently address mitochondrial failure and neuroinflammation. The agents discussed herein, whether gasses, peptides, natural compounds, or synthetic inhibitors, converge on critical nodes within the pathogenic network of SAE. The most promising approaches are those that not only rescue mitochondrial function but also break the vicious cycle of inflammation, such as H_2_ therapy and SS-31. Future work should focus on translating these robust preclinical findings into clinical trials and exploring potential synergistic effects of combination therapies.

### 6.6 Critical evaluation and clinical translation challenges

Despite the promising preclinical evidence for various therapeutic strategies targeting the mitochondrial-neuroinflammatory axis in SAE, several challenges must be addressed before these approaches can be translated into clinical practice. A balanced evaluation of their advantages and limitations is essential for guiding future research and clinical trial design.

Molecular H_2_ offers the advantages of high bioavailability, excellent safety profile, and pleiotropic effects (antioxidant, anti-inflammatory, and bioenergetic) (Cui et al., 2024). However, its rapid systemic clearance necessitates continuous or repeated administration, and the optimal dosage, timing, and route (inhalation vs. H_2_-rich saline) remain to be standardized. The lack of potent, target-specific effects may also limit its efficacy in severe SAE.

DCA effectively shifts metabolism toward oxidative phosphorylation and reduces Drp1-mediated fission (Huang et al., 2025). However, its clinical use is limited by potential neurotoxicity (e.g., reversible peripheral neuropathy) with prolonged administration, and its narrow therapeutic window requires careful dose optimization.

Mitochondria-targeted agents such as SS-31 and Mdivi-1 offer high specificity for mitochondrial structures or dynamics regulators, demonstrating robust neuroprotection in preclinical models (Dai et al., 2025; Wu et al., 2015). Nonetheless, their clinical translation is hindered by challenges related to peptide stability and delivery (SS-31), potential off-target effects (Mdivi-1), and limited data on long-term safety and pharmacokinetics in humans.

Natural compounds (e.g., (-)-epicatechin, malvidin, STS) are attractive due to their multi-target effects, oral bioavailability, and favorable safety profiles derived from dietary sources (Zhao et al., 2022; Ling et al., 2022; Song et al., 2026). However, their relatively low potency, variable absorption, and lack of standardized formulations pose significant hurdles. Moreover, the mechanistic pleiotropy makes it difficult to attribute therapeutic effects to specific pathways, complicating regulatory approval.

Specific pharmacological inhibitors (e.g., MCC950, P2X7R inhibitors, Fgr inhibitors) provide the advantage of precise pathway modulation, enabling hypothesis-driven intervention (Liu et al., 2023; Wang et al., 2022; Song et al., 2026). However, concerns regarding immune suppression, off-target toxicity, and the evolutionary conservation of targets (e.g., NLRP3) necessitate rigorous safety evaluation. Additionally, the high cost of developing novel small molecules may limit accessibility.

FMT represents a novel approach targeting the gut-brain axis, with the unique advantage of restoring microbial diversity and metabolite profiles (Zhang et al., 2022). Nevertheless, FMT carries inherent risks, including pathogen transmission, variable donor efficacy, and lack of standardization. Its invasive nature and patient acceptability also remain concerns.

In summary, while each therapeutic strategy offers distinct mechanistic advantages, successful clinical translation will require: (1) rigorous pharmacokinetic and toxicity profiling; (2) identification of optimal therapeutic windows; (3) development of biomarkers for patient stratification; and (4) consideration of cost-effectiveness and scalability. Future research should also explore rational combination therapies that synergistically target multiple nodes of the mitochondrial-neuroinflammatory axis while minimizing individual agent toxicities.

### 6.7 Nanomedicine and biomimetic approaches

Recent advances in nanomedicine have opened innovative avenues for targeted intervention in SAE, offering the potential to overcome limitations of conventional therapies such as poor BBB penetration, non-specific distribution, and rapid clearance.

Biomimetic nanomodulators represent a particularly promising strategy. Qu et al. (2024) developed a biomimetic nanomodulator that integrates the properties of natural cell membranes or enzyme-mimicking nanoparticles to simultaneously scavenge reactive oxygen species and neutralize pro-inflammatory cytokines in the brain microenvironment. By mimicking natural detoxification mechanisms, this nanoplatform efficiently penetrated the impaired BBB and accumulated in activated microglia and neurons. It concurrently reduced mitochondrial ROS production and inhibited the NLRP3 inflammasome pathway, thereby breaking the vicious cycle between oxidative stress and neuroinflammation in a murine SAE model (Qu et al., 2024).

An even more sophisticated approach involves the combination of nanomaterials with cell-based therapies. Ben Nun et al. (2026) engineered “polyphenolic nanobattery-charged” adoptive macrophages, where macrophages are loaded with polyphenolic nanoparticles before being transferred into the host. This strategy aims to pre-program the metabolic and anti-inflammatory state of the transferred cells, enhancing their resilience and therapeutic efficacy in the septic microenvironment. The polyphenolic nanoparticles act as intracellular “batteries,” continuously modulating the macrophage’s redox balance and inflammatory phenotype (Ben Nun et al., 2026). While this specific study focuses on systemic sepsis immunotherapy, its successful application could pave the way for engineering microglia or infiltrating macrophages to adopt a sustained pro-repair (M2-like) phenotype in the CNS, offering a powerful new avenue for SAE treatment.

Other nanomaterial-based strategies under investigation for SAE include cerium oxide nanoparticles (which exhibit potent ROS-scavenging enzyme-mimetic activities), mesoporous silica nanoparticles for targeted drug delivery across the BBB, and lipid-based nanoparticles for delivering nucleic acid therapeutics (e.g., siRNA, miRNA) to modulate microglial activation (Ling et al., 2022; Zhang et al., 2025; Ding et al., 2022). These platforms offer the advantages of tunable surface modification, controlled release kinetics, and the ability to co-deliver multiple therapeutic agents.

Despite their promise, the clinical translation of nanomaterials for SAE faces significant challenges, including potential long-term toxicity, accumulation in off-target organs, and the complexity of manufacturing under good manufacturing practice conditions. Future research should focus on optimizing nanoparticle design for enhanced BBB penetration, biodegradability, and target specificity, as well as rigorous preclinical safety evaluation in clinically relevant animal models.

## 7 Conclusion and future perspectives

This comprehensive review underscores that mitochondrial dysfunction and neuroinflammation are not parallel but intimately interconnected processes that collectively drive the pathogenesis of SAE. The core findings can be summarized as follows: (1) mitochondrial dynamics are profoundly disrupted in SAE, with excessive Drp1-mediated fission and impaired fusion leading to bioenergetic failure, oxidative stress, and activation of mitochondria-dependent cell death pathways (apoptosis, pyroptosis, ferroptosis); (2) microglial activation, particularly through the NLRP3 inflammasome, creates a self-perpetuating cycle of neuroinflammation that exacerbates mitochondrial damage and synaptic loss; (3) BBB disruption, driven by endothelial mitochondrial dysfunction and tight junction disassembly, facilitates the entry of neurotoxic mediators into the brain parenchyma; (4) the gut-brain axis and cerebral metabolomic alterations critically modulate mitochondrial function and inflammatory responses; and (5) emerging therapeutic strategies targeting this mitochondrial-neuroinflammatory axis—including molecular hydrogen, mitochondria-targeted peptides, natural compounds, and specific inhibitors—have demonstrated robust preclinical efficacy by restoring mitochondrial homeostasis and resolving neuroinflammation.

Despite these advances, the transition from preclinical promise to clinical reality requires a more refined and forward-looking research agenda. We propose the following specific directions for future investigation:

### 7.1 Mechanism-based drug development targeting specific molecular nodes

While multi-target agents offer pleiotropic benefits, the development of highly selective compounds against well-validated targets—such as Drp1 (e.g., next-generation inhibitors beyond Mdivi-1), the NLRP3 inflammasome (e.g., third-generation inhibitors with improved bioavailability), or the PGC-1α pathway (e.g., specific activators)—remains a priority. Structure-based drug design and high-throughput screening campaigns are needed to identify molecules with optimal pharmacokinetic profiles, BBB penetrance, and minimal off-target effects. Furthermore, exploring protein–protein interaction interfaces (e.g., Drp1-Fis1) as drug targets may yield higher specificity than targeting enzymatic active sites.

### 7.2 Rational design of multi-target combination therapy strategies

Given the multifaceted nature of SAE, a single-agent approach may be insufficient to fully reverse the pathological cascade. Future studies should systematically evaluate synergistic combinations that concurrently target distinct yet interconnected nodes. For example, combining a mitochondrial protectant (e.g., SS-31) with an NLRP3 inflammasome inhibitor (e.g., MCC950) and a gut microbiome modulator (e.g., probiotics or FMT) could simultaneously address bioenergetic failure, inflammation amplification, and systemic metabolic dysregulation. Preclinical trials employing factorial design or response-surface methodology will be essential to identify optimal dose ratios and sequence of administration.

### 7.3 Development of personalized treatment plans based on multi-omics technologies

The marked heterogeneity of SAE among patients, in terms of underlying sepsis etiology, genetic susceptibility, immune status, and gut microbiota composition, calls for a precision medicine approach. Future research should leverage integrated multi-omics analyses (genomics, epigenomics, transcriptomics, proteomics, metabolomics, and metagenomics) to stratify patients into distinct endotypes. For instance, metabolomic profiling of cerebrospinal fluid or plasma could identify patients with predominant mitochondrial dysfunction (e.g., low ATP, high mtDNA) versus those with hyperinflammation (e.g., elevated IL-1β, IL-18). Such stratification would enable targeted assignment of mechanism-based therapies. Additionally, longitudinal multi-omics monitoring could identify dynamic biomarkers for treatment response, enabling real-time therapeutic adjustment.

### 7.4 Overcoming translational barriers through advanced preclinical models and clinical trial design

The current over-reliance on young, healthy animal models with uniform septic insults limits the predictive validity of preclinical findings. Future studies should incorporate clinically relevant variables such as age, comorbidities, and polypharmacy, and utilize models that recapitulate the chronic cognitive sequelae of SAE. In parallel, adaptive clinical trial designs (e.g., platform trials, umbrella trials) that allow for simultaneous evaluation of multiple investigational agents in biomarker-defined patient subgroups could accelerate the identification of effective therapies.

In conclusion, the mitochondrial-neuroinflammatory axis represents a paradigm-shifting framework for understanding SAE pathophysiology and developing effective interventions. By pursuing mechanism-driven drug discovery, rational combination strategies, and personalized multi-omics-guided approaches, the field is poised to translate fundamental insights into tangible improvements in neurological outcomes for the millions of sepsis survivors worldwide.

## References

[ref1] Andonegui G. Zelinski E. L. Schubert C. L. Knight D. Craig L. A. Winston B. W. . (2018). Targeting inflammatory monocytes in sepsis-associated encephalopathy and long-term cognitive impairment. JCI Insight 3:99364. doi: 10.1172/jci.insight.99364, 29720578 PMC6012517

[ref2] Ben Nun U. Yesodi D. Lemcoff N. Gordon G. Katz A. Shelonchik O. . (2026). Photothermal nanocomposite hydrogels enable precise nucleic acid manipulation and separation. Cell Biomater.:100406. doi: 10.1016/j.celbio.2026.100406

[ref3] Bonfante S. Joaquim L. Fileti M. E. Giustina A. D. de Souza Goldim M. P. Danielski L. G. . (2021). Stanniocalcin 1 inhibits the inflammatory response in microglia and protects against Sepsis-associated encephalopathy. Neurotox. Res. 39, 119–132. doi: 10.1007/s12640-020-00293-y, 33025358

[ref4] Bourguinat C. Kamgno J. Boussinesq M. Mackenzie C. D. Prichard R. K. Geary T. G. (2010). Analysis of the mdr-1 gene in patients co-infected with *Onchocerca volvulus* and *Loa loa* who experienced a post-ivermectin serious adverse event. Am J Trop Med Hyg 83, 28–32. doi: 10.4269/ajtmh.2010.09-0734, 20595473 PMC2912571

[ref5] Catarina A. V. Branchini G. Bettoni L. de Oliveira J. R. Nunes F. B. (2021). Sepsis-associated encephalopathy: from pathophysiology to Progress in experimental studies. Mol. Neurobiol. 58, 2770–2779. doi: 10.1007/s12035-021-02303-2, 33495934

[ref6] Cui W. Chen J. Yu F. Liu W. He M. (2021). GYY4137 protected the integrity of the blood-brain barrier via activation of the Nrf2/ARE pathway in mice with sepsis. FASEB J. 35:e21710. doi: 10.1096/fj.202100074R, 34143548

[ref7] Cui Y. Meng S. Zhang N. Liu J. Zheng L. Ma W. . (2024). High-concentration hydrogen inhalation mitigates sepsis-associated encephalopathy in mice by improving mitochondrial dynamics. CNS Neurosci. Ther. 30:e70021. doi: 10.1111/cns.70021, 39258790 PMC11388582

[ref8] Dai L. Gu S. Zhang Y. Ma S. Wang P. Zhang J. . (2025). Mitochondrial fission inhibitor Mdivi-1 alleviates lipopolysaccharide-induced parvalbumin interneurons dysregulation and cognitive impairments in a mouse model of sepsis-associated encephalopathy. Front. Pharmacol. 16:1525028. doi: 10.3389/fphar.2025.1525028, 40575778 PMC12198682

[ref9] Dela Cruz C. S. Kang M. J. (2018). Mitochondrial dysfunction and damage associated molecular patterns (DAMPs) in chronic inflammatory diseases. Mitochondrion 41, 37–44. doi: 10.1016/j.mito.2017.12.001, 29221810 PMC5988941

[ref10] Ding H. Li Y. Chen S. Wen Y. Zhang S. Luo E. . (2022). Fisetin ameliorates cognitive impairment by activating mitophagy and suppressing neuroinflammation in rats with sepsis-associated encephalopathy. CNS Neurosci. Ther. 28, 247–258. doi: 10.1111/cns.13765, 34837343 PMC8739041

[ref11] Duan H. Yang X. Cai S. Zhang L. Qiu Z. Wang J. . (2024). Nrf2 mitigates sepsis-associated encephalopathy-induced hippocampus ferroptosis via modulating mitochondrial dynamic homeostasis. Int. Immunopharmacol. 143:113331. doi: 10.1016/j.intimp.2024.113331, 39396427

[ref12] Dumbuya J. S. Chen X. du J. Li S. Liang L. Xie H. . (2023). Hydrogen-rich saline regulates NLRP3 inflammasome activation in sepsis-associated encephalopathy rat model. Int. Immunopharmacol. 123:110758. doi: 10.1016/j.intimp.2023.110758, 37556997

[ref13] Dumbuya J. S. Li S. Liang L. Chen Y. du J. Zeng Q. (2022). Effects of hydrogen-rich saline in neuroinflammation and mitochondrial dysfunction in rat model of sepsis-associated encephalopathy. J. Transl. Med. 20:546. doi: 10.1186/s12967-022-03746-4, 36435787 PMC9701391

[ref14] Fu Q. Zhang Y. B. Shi C. X. Jiang M. Lu K. Fu Z. H. . (2024). GSDMD/Drp1 signaling pathway mediates hippocampal synaptic damage and neural oscillation abnormalities in a mouse model of sepsis-associated encephalopathy. J. Neuroinflammation 21:96. doi: 10.1186/s12974-024-03084-w, 38627764 PMC11020266

[ref15] Guan S. Sun L. Wang X. Huang X. Luo T. (2023). Propofol inhibits neuroinflammation and metabolic reprogramming in microglia in vitro and in vivo. Front. Pharmacol. 14:1161810. doi: 10.3389/fphar.2023.1161810, 37383725 PMC10293632

[ref16] Haileselassie B. Joshi A. U. Minhas P. S. Mukherjee R. Andreasson K. I. Mochly-Rosen D. (2020). Mitochondrial dysfunction mediated through dynamin-related protein 1 (Drp1) propagates impairment in blood brain barrier in septic encephalopathy. J. Neuroinflammation 17:36. doi: 10.1186/s12974-019-1689-8, 31987040 PMC6986002

[ref17] Han Y. Xie X. Qiu J. Tang Y. Song Z. Li W. . (2025). Early prediction of sepsis associated encephalopathy in elderly ICU patients using machine learning models: a retrospective study based on the MIMIC-IV database. Front. Cell. Infect. Microbiol. 15:1545979. doi: 10.3389/fcimb.2025.1545979, 40313459 PMC12043699

[ref18] Hao S. Luo J. Yuan S. Chen W. Zhang X. Zhao C. . (2025). The DRP1 inhibitory peptide P110 provides neuroprotection after subarachnoid hemorrhage by suppressing neuronal apoptosis and stabilizing the blood-brain barrier. Free Radic. Biol. Med. 240, 1–14. doi: 10.1016/j.freeradbiomed.2025.08.011, 40784584

[ref19] Hong Y. Chen P. Gao J. Lin Y. Chen L. Shang X. (2023). Sepsis-associated encephalopathy: from pathophysiology to clinical management. Int. Immunopharmacol. 124:110800. doi: 10.1016/j.intimp.2023.110800, 37619410

[ref20] Hong C. Wang L. Zhou X. Zou L. Xiang X. Deng H. . (2025). Protective effects of Mdivi-1 on cognition disturbance following Sepsis in mice via alleviating microglia activation and polarization. CNS Neurosci. Ther. 31:e70149. doi: 10.1111/cns.70149, 39791542 PMC11719124

[ref21] Hu Y. Bi Y. Yao D. Wang P. Li Y. (2019). Omi/HtrA2 protease associated cell apoptosis participates in blood-brain barrier dysfunction. Front. Mol. Neurosci. 12:48. doi: 10.3389/fnmol.2019.00048, 30853894 PMC6395387

[ref22] Huang L. Chen J. Li X. Huang M. Liu J. Qin N. . (2022). Polydatin improves Sepsis-associated encephalopathy by activating Sirt1 and reducing p38 phosphorylation. J. Surg. Res. 276, 379–393. doi: 10.1016/j.jss.2022.03.008, 35447391

[ref23] Huang C. T. Wang Y. C. Lin S. C. Lai Y. C. Chen S. H. Feng S. T. . (2026). Impact of gut microbiota alterations on mitochondrial bioenergetics in cortical astrocytes and sensorimotor impairment in a rat model of lipopolysaccharide-associated encephalopathy. Shock 65, 316–328. doi: 10.1097/SHK.0000000000002637 40550557 PMC12863598

[ref24] Huang X. Ye C. Zhao X. Tong Y. Lin W. Huang Q. . (2023). TRIM45 aggravates microglia pyroptosis via Atg5/NLRP3 axis in septic encephalopathy. J. Neuroinflammation 20:284. doi: 10.1186/s12974-023-02959-8, 38037161 PMC10688018

[ref25] Huang L. Zhan D. Xing Y. Yan Y. Li Q. Zhang J. . (2023). FGL2 deficiency alleviates maternal inflammation-induced blood-brain barrier damage by blocking PI3K/NF-κB mediated endothelial oxidative stress. Front. Immunol. 14:1157027. doi: 10.3389/fimmu.2023.1157027, 37051251 PMC10083319

[ref26] Huang X. Zheng Y. Wang N. Zhao M. Liu J. Lin W. . (2025). Dichloroacetate prevents Sepsis associated encephalopathy by inhibiting microglia Pyroptosis through PDK4/NLRP3. Inflammation 48, 1159–1175. doi: 10.1007/s10753-024-02105-3, 39177920 PMC12234639

[ref27] U-Pathi J. Yeh Y. C. Chen C. W. Owaga E. E. Hsieh R. H. (2023). Relationship between aspartame-induced cerebral cortex injury and oxidative stress, inflammation, mitochondrial dysfunction, and apoptosis in Sprague Dawley rats. Antioxidants (Basel) 13:2. doi: 10.3390/antiox13010002 38275622 PMC10812821

[ref28] Jing G. Gong H. Wang H. Zuo J. Wu D. Liu H. . (2025). OTUD1 exacerbates sepsis-associated encephalopathy by promoting HK2 mitochondrial release to drive microglia pyroptosis. J. Neuroinflammation 22:154. doi: 10.1186/s12974-025-03480-w, 40500776 PMC12153095

[ref29] Kan Y. Wang H. Lin H. Li Y. Pei S. Cui Y. . (2025). Transcript and lipid profile alterations in astrocyte-neuron mitochondrial transfer under lipopolysaccharide exposure: an in vitro study. J. Neurochem. 169:e70003. doi: 10.1111/jnc.70003, 39902645 PMC11791887

[ref30] Kikuchi D. S. Campos A. C. P. Qu H. Forrester S. J. Pagano R. L. Lassègue B. . (2019). Poldip2 mediates blood-brain barrier disruption in a model of sepsis-associated encephalopathy. J. Neuroinflammation 16:241. doi: 10.1186/s12974-019-1575-4, 31779628 PMC6883676

[ref31] Kiyuna L. A. Albuquerque R. P. Chen C. H. Mochly-Rosen D. Ferreira J. C. B. (2018). Targeting mitochondrial dysfunction and oxidative stress in heart failure: challenges and opportunities. Free Radic. Biol. Med. 129, 155–168. doi: 10.1016/j.freeradbiomed.2018.09.019, 30227272 PMC6309415

[ref32] Kleele T. Rey T. Winter J. Zaganelli S. Mahecic D. Perreten Lambert H. . (2021). Distinct fission signatures predict mitochondrial degradation or biogenesis. Nature 593, 435–439. doi: 10.1038/s41586-021-03510-6, 33953403

[ref33] Ko M. S. Yun J. Y. Baek I. J. Jang J. E. Hwang J. J. Lee S. E. . (2021). Mitophagy deficiency increases NLRP3 to induce brown fat dysfunction in mice. Autophagy 17, 1205–1221. doi: 10.1080/15548627.2020.1753002, 32400277 PMC8143238

[ref34] Kobayashi T. Uchino H. Elmér E. Ogihara Y. Fujita H. Sekine S. . (2022). Disease outcome and brain metabolomics of cyclophilin-D knockout mice in Sepsis. Int. J. Mol. Sci. 23:961. doi: 10.3390/ijms23020961, 35055146 PMC8779771

[ref35] Lei Y. Zhou R. Sun X. Tang F. Gao H. Chen L. . (2021). The pannexin-1 channel regulates pyroptosis through autophagy in a mouse model of sepsis-associated encephalopathy. Ann Transl Med 9:1802. doi: 10.21037/atm-21-6579, 35071496 PMC8756244

[ref36] Li Y. Feng Y. F. Liu X. T. Li Y. C. Zhu H. M. Sun M. R. . (2021). Songorine promotes cardiac mitochondrial biogenesis via Nrf2 induction during sepsis. Redox Biol. 38:101771. doi: 10.1016/j.redox.2020.101771, 33189984 PMC7674615

[ref37] Li J. Jia Q. Yang L. Wu Y. Peng Y. du L. . (2025). Sepsis-associated encephalopathy: mechanisms, diagnosis, and treatments update. Int. J. Biol. Sci. 21, 3214–3228. doi: 10.7150/ijbs.102234, 40384873 PMC12080397

[ref38] Ling J. Wu Y. Zou X. Chang Y. Li G. Fang M. (2022). (-)-epicatechin reduces neuroinflammation, protects mitochondria function, and prevents cognitive impairment in Sepsis-associated encephalopathy. Oxidative Med. Cell. Longev. 2022:2657713. doi: 10.1155/2022/2657713, 35656027 PMC9155907

[ref39] Liu Y. Guo L. Zhang G. Sun W. Yang X. (2024). Nogo-a exacerbates sepsis-associated encephalopathy by modulating microglial SHP-2/NLRP3 balance and inducing ROS and M1 polarization. Biomol. Biomed. 25, 210–225. doi: 10.17305/bb.2024.10822, 39151100 PMC11647263

[ref40] Liu S. Liu Z. Wu G. Ye H. Wu Z. Yang Z. . (2024). Assessment of sepsis-associated encephalopathy by quantitative magnetic resonance spectroscopy in a rat model of cecal ligation and puncture. Heliyon 10:e26836. doi: 10.1016/j.heliyon.2024.e26836, 38434271 PMC10906417

[ref41] Liu Y. Yang H. Luo N. Fu Y. Qiu F. Pan Z. . (2023). An Fgr kinase inhibitor attenuates sepsis-associated encephalopathy by ameliorating mitochondrial dysfunction, oxidative stress, and neuroinflammation via the SIRT1/PGC-1α signaling pathway. J. Transl. Med. 21:486. doi: 10.1186/s12967-023-04345-7, 37475042 PMC10360347

[ref42] Liu H. Zhang T. Zhang L. Zhong Y. (2025). Neuroinflammatory mechanisms of adult sepsis-associated encephalopathy: implications for blood-brain barrier disruption and oxidative stress. Diagnostics (Basel) 15:873. doi: 10.3390/diagnostics15070873, 40218223 PMC11988331

[ref43] Luo Y. Xu D. Yu C. (2025). Research progress on sepsis-associated encephalopathy by inhibiting pyroptosis. Gene 961:149560. doi: 10.1016/j.gene.2025.149560, 40355013

[ref44] Ma Y. She X. Liu Y. Qin X. (2024). MSC-derived exosomal miR-140-3p improves cognitive dysfunction in sepsis-associated encephalopathy by HMGB1 and S-lactoylglutathione metabolism. Commun. Biol. 7:562. doi: 10.1038/s42003-024-06236-z, 38734709 PMC11088640

[ref45] Meng D. Yin G. Chen S. Zhang X. Yu W. Wang L. . (2024). Diosgenin attenuates nonalcoholic hepatic steatosis through the hepatic SIRT1/PGC-1α pathway. Eur. J. Pharmacol. 977:176737. doi: 10.1016/j.ejphar.2024.176737, 38866362

[ref46] Modafferi S. Molinari F. Interdonato L. Fusco R. Impellizzeri D. Siracusa R. . (2024). Change in Nfkb/Nrf2/Bax levels by high monomeric polyphenols berries extract (HMPBE) in acute and chronic secondary brain damage. Cell. Physiol. Biochem. 58, 548–570. doi: 10.33594/000000731, 39370950

[ref47] Moraes C. A. Hottz E. D. dos Santos Ornellas D. Adesse D. de Azevedo C. T. d’Avila J. C. . (2023). Microglial NLRP3 inflammasome induces excitatory synaptic loss through IL-1β-enriched microvesicle release: implications for Sepsis-associated encephalopathy. Mol. Neurobiol. 60, 481–494. doi: 10.1007/s12035-022-03067-z, 36280654

[ref48] Niu X. Wei P. Zhu M. Zheng H. Kang D. Chen Q. . (2025). Iron single atom enzyme-mediated hydrogen sulfide delivery amplifies reactive oxygen species cascade to induce ferroptosis susceptibility. Mater Today Bio 34:102184. doi: 10.1016/j.mtbio.2025.102184, 40838212 PMC12363588

[ref49] Nunnari J. Suomalainen A. (2012). Mitochondria: in sickness and in health. Cell 148, 1145–1159. doi: 10.1016/j.cell.2012.02.035, 22424226 PMC5381524

[ref50] Pan S. Lv Z. Wang R. Shu H. Yuan S. Yu Y. . (2022). Sepsis-induced brain dysfunction: pathogenesis, diagnosis, and treatment. Oxidative Med. Cell. Longev. 2022:1328729. doi: 10.1155/2022/1328729, 36062193 PMC9433216

[ref51] Qu H. Wu J. Pan Y. Abdulla A. Duan Z. Cheng W. . (2024). Biomimetic Nanomodulator regulates oxidative and inflammatory stresses to treat Sepsis-associated encephalopathy. ACS Nano 18, 28228–28245. doi: 10.1021/acsnano.4c08157, 39367850

[ref52] Song S. Ding L. Huang Q. Zhang Z. Cao L. Yin K. . (2026). Qingqi Liangying formula inhibits brain endothelial cell pyroptosis via NLRP3/caspase-1/GSDMD in sepsis-associated encephalopathy. Phytomedicine 150:157391. doi: 10.1016/j.phymed.2025.157391, 41442993

[ref53] Song Y. Lin W. Yuan H. Hu H. Wu S. Lu Y. . (2026). Sodium tanshinone IIA sulfonate alleviates neuroinflammation-induced damage to hippocampal neurons by activating SIRT1 in mice with sepsis-associated encephalopathy. J. Ethnopharmacol. 355:120731. doi: 10.1016/j.jep.2025.120731 41072780

[ref54] Tang Z. Li R. Guo X. Wang Z. Wu J. (2025). Regulation of blood-brain barrier integrity by brain microvascular endothelial cells in ischemic stroke: a therapeutic opportunity. Eur. J. Pharmacol. 996:177553. doi: 10.1016/j.ejphar.2025.177553, 40147580

[ref55] von der Malsburg A. Sapp G. M. Zuccaro K. E. von Appen A. Moss F. R. III Kalia R. . (2023). Structural mechanism of mitochondrial membrane remodelling by human OPA1. Nature 620, 1101–1108. doi: 10.1038/s41586-023-06441-6, 37612504 PMC10875962

[ref56] Wang Y. Chen Z. Zhang Y. Fang S. Zeng Q. (2014). Mitochondrial biogenesis of astrocytes is increased under experimental septic conditions. Chin. Med. J. 127, 1837–1842. doi: 10.3760/cma.j.issn.0366-6999.20131934, 24824241

[ref57] Wang P. Hu Y. Yao D. Li Y. (2018). Omi/HtrA2 regulates a mitochondria-dependent apoptotic pathway in a murine model of septic encephalopathy. Cell. Physiol. Biochem. 49, 2163–2173. doi: 10.1159/000493819, 30286467

[ref58] Wang P. Liang L. Ge Q. Liu S. Yang Z. Jiang L. (2024). Dichloroacetate attenuates brain injury through inhibiting neuroinflammation and mitochondrial fission in a rat model of sepsis-associated encephalopathy. Int. Immunopharmacol. 140:112840. doi: 10.1016/j.intimp.2024.112840, 39106713

[ref59] Wang S. Liu Z. Li R. Wang L. Wu Y. Zhang W. . (2025). Acetaldehyde dehydrogenase 2 attenuates lipopolysaccharide -induced endothelial barrier damage by inhibiting mitochondrial fission in sepsis-associated encephalopathy. Eur. J. Pharmacol. 997:177468. doi: 10.1016/j.ejphar.2025.177468, 40054720

[ref60] Wang K. Sun M. Juan Z. Zhang J. Sun Y. Wang G. . (2022). The improvement of sepsis-associated encephalopathy by P2X7R inhibitor through inhibiting the Omi/HtrA2 apoptotic signaling pathway. Behav. Neurol. 2022:3777351. doi: 10.1155/2022/3777351, 35126784 PMC8813303

[ref61] Wang J. Zhu Q. Wang Y. Peng J. Shao L. Li X. (2022). Irisin protects against sepsis-associated encephalopathy by suppressing ferroptosis via activation of the Nrf2/GPX4 signal axis. Free Radic. Biol. Med. 187, 171–184. doi: 10.1016/j.freeradbiomed.2022.05.023 35660523

[ref62] Wu H. Li N. Peng S. Fu H. Hu Z. Su L. (2024). Maresin1 improves hippocampal neuroinflammation and cognitive function in septic rats by activating the SLC7A11/GPX4 ferroptosis signaling pathway. Int. Immunopharmacol. 131:111792. doi: 10.1016/j.intimp.2024.111792, 38484667

[ref63] Wu J. Zhang M. Hao S. Jia M. Ji M. Qiu L. . (2015). Mitochondria-targeted peptide reverses mitochondrial dysfunction and cognitive deficits in Sepsis-associated encephalopathy. Mol. Neurobiol. 52, 783–791. doi: 10.1007/s12035-014-8918-z, 25288156

[ref64] Xie K. Wang Y. Yin L. Wang Y. Chen H. Mao X. . (2021). Hydrogen gas alleviates sepsis-induced brain injury by improving mitochondrial biogenesis through the activation of PGC-α in mice. Shock 55, 100–109. doi: 10.1097/SHK.0000000000001594 32590694

[ref65] Xie K. Zhang Y. Wang Y. Meng X. Yu Y. Chen H. (2020). Hydrogen attenuates sepsis-associated encephalopathy by NRF2 mediated NLRP3 pathway inactivation. Inflamm. Res. 69, 697–710. doi: 10.1007/s00011-020-01347-9, 32350570

[ref66] Xiong F. Wang C. Lu J. Bai G. Zhou D. Ling J. (2024). 4-PBA exerts brain-protective effects against sepsis-associated encephalopathy in a mouse model of sepsis. Exp. Neurol. 375:114738. doi: 10.1016/j.expneurol.2024.114738, 38395217

[ref67] Yan C. Duanmu X. Zeng L. Liu B. Song Z. (2019). Mitochondrial DNA: distribution, mutations, and elimination. Cells 8:379. doi: 10.3390/cells8040379, 31027297 PMC6523345

[ref68] Yan X. Yang K. Xiao Q. Hou R. Pan X. Zhu X. (2022). Central role of microglia in sepsis-associated encephalopathy: from mechanism to therapy. Front. Immunol. 13:929316. doi: 10.3389/fimmu.2022.929316, 35958583 PMC9361477

[ref69] Yang X. Duan H. Li S. Zhang J. Dong L. Ding J. . (2024). Yap1 alleviates sepsis associated encephalopathy by inhibiting hippocampus ferroptosis via maintaining mitochondrial dynamic homeostasis. J. Cell. Mol. Med. 28:e70156. doi: 10.1111/jcmm.70156, 39400418 PMC11472648

[ref70] Zhan Y. Zhang L. Sun J. Yao H. Chen J. Tian M. (2024). ADSC-derived exosomes provide neuroprotection in sepsis-associated encephalopathy by regulating hippocampal pyroptosis. Exp. Neurol. 380:114900. doi: 10.1016/j.expneurol.2024.114900, 39059736

[ref71] Zhang Y. Chen J. Wu H. Li L. Yang X. Lai K. . (2023). Hydrogen regulates mitochondrial quality to protect glial cells and alleviates sepsis-associated encephalopathy by Nrf2/YY1 complex promoting HO-1 expression. Int. Immunopharmacol. 118:110009. doi: 10.1016/j.intimp.2023.110009, 36963264

[ref72] Zhang Y. Fu Q. Ruan J. Shi C. Lu W. Wu J. . (2023). Dexpramipexole ameliorates cognitive deficits in sepsis-associated encephalopathy through suppressing mitochondria-mediated pyroptosis and apoptosis. Neuroreport 34, 220–231. doi: 10.1097/WNR.0000000000001882, 36719835 PMC10516177

[ref73] Zhang L. Jiang Y. Deng S. Mo Y. Huang Y. Li W. . (2021). S100B/RAGE/ceramide signaling pathway is involved in sepsis-associated encephalopathy. Life Sci. 277:119490. doi: 10.1016/j.lfs.2021.119490, 33862114

[ref74] Zhang S. Luo N. Wu H. Chen J. Jiang Y. Xiao L. . (2025). Capsaicin attenuates sepsis-associated encephalopathy by inhibiting neuroinflammation and apoptosis whilst activating mitophagy through the BNIP3/NIX pathway. Mol. Med. Rep. 32, 1–13. doi: 10.3892/mmr.2025.13686, 40970326 PMC12461237

[ref75] Zhang H. Xu J. Wu Q. Fang H. Shao X. Ouyang X. . (2022). Gut microbiota mediates the susceptibility of mice to Sepsis-associated encephalopathy by butyric acid. J. Inflamm. Res. 15, 2103–2119. doi: 10.2147/JIR.S350566, 35386224 PMC8977350

[ref76] Zhao P. Li X. Yang Q. Lu Y. Wang G. Yang H. . (2022). Malvidin alleviates mitochondrial dysfunction and ROS accumulation through activating AMPK-α/UCP2 axis, thereby resisting inflammation and apoptosis in SAE mice. Front. Pharmacol. 13:1038802. doi: 10.3389/fphar.2022.1038802, 36699054 PMC9868257

[ref77] Zhao Q. Liu G. Ding Q. Zheng F. Shi X. Lin Z. . (2024). The ROS/TXNIP/NLRP3 pathway mediates LPS-induced microglial inflammatory response. Cytokine 181:156677. doi: 10.1016/j.cyto.2024.156677, 38896955

[ref78] Zhong H. Liu T. Shang Y. Huang C. Pan S. (2024). Breaking the vicious cycle: targeting the NLRP3 inflammasome for treating sepsis-associated encephalopathy. Biomed. Pharmacother. 177:117042. doi: 10.1016/j.biopha.2024.117042, 39004064

[ref79] Zhong L. Ren X. Ai Y. Liu Z. (2023). SS-31 improves cognitive function in Sepsis-associated encephalopathy by inhibiting the Drp1-NLRP3 inflammasome activation. NeuroMolecular Med. 25, 230–241. doi: 10.1007/s12017-022-08730-1, 36333543

[ref80] Zhu L. Ma L. du X. Jiang Y. Gao J. Fan Z. . (2024). M2 microglia-derived exosomes protect against glutamate-induced HT22 cell injury via Exosomal miR-124-3p. Mol. Neurobiol. 61, 7845–7861. doi: 10.1007/s12035-024-04075-x, 38433165 PMC11415474

